# Snake Venomics of the Arboreal Talamancan Palm-Pitviper, *Bothriechis nubestris*, Provides Clues on the Origin of a Phenotypic Dichotomy between Type‑I and Type-II Venoms

**DOI:** 10.1021/acs.jproteome.4c01041

**Published:** 2025-04-25

**Authors:** Johelen Chacón, Stephanie Chaves-Araya, Gianni Mena, Arturo Chang-Castillo, Cecilia Díaz, Fabián Bonilla, Mahmood Sasa, Juan J. Calvete, Libia Sanz, José María Gutiérrez, Bruno Lomonte, Julián Fernández

**Affiliations:** † Instituto Clodomiro Picado, Facultad de Microbiología, 27915 Universidad de Costa Rica, San José 11501, Costa Rica; ‡ Museo de Zoología, Centro de Investigaciones en Biodiversidad y Ecología Tropical, 27915 Universidad de Costa Rica, San José 11501, Costa Rica; § Laboratorio de Venómica Evolutiva y Traslacional, 54426 Instituto de Biomedicina de Valencia, Consejo Superior de Investigaciones Científicas, Jaume Roig 11, 46010 Valencia, Spain

**Keywords:** Snake venomics, Talamancan palm-pitviper, *Bothriechis nubestris*, ICP antivenom, venom type-I, type-II dichotomy, miRNA

## Abstract

We report the biochemical and proteomic characterization of the venom of the arboreal Talamancan palm-pitviper, *Bothriechis nubestris*, a species endemic to north and central parts of the Costa Rican Cordillera de Talamanca, at 2100 to 3000 m above sea level. The Talamancan palm-pitviper venom arsenal comprised the translated products of 26 unique transcripts into 10 toxin classes, where metalloproteinases PIIa and PIII represent the dominant components. *In vitro*, the venom proteolyzed azocasein and gelatin but showed no phospholipase A_2_ or human plasma coagulant activities. *In vivo*, *B. nubestris* venom exhibited an intravenous median lethal dose (LD_50_) of 21.5 (95% confidence interval: 15.6–29.5) μg/mouse, and a minimum hemorrhagic dose (MHD) of 1.85 μg. PoliVal-ICP antivenom neutralized the venom’s lethal activity with a potency of 9.7 mg of venom/g of antivenom and significantly reduced the hemorrhagic effect. Comparison of venom gland transcriptomes and venom proteomes of *B. nubestris* and its closest congeneric relative, *B. nigroviridis*, revealed that highly conserved venom gland transcriptomes are differentially processed by each species to produce divergent (type-I vs type-II, respectively) venoms. This phenomenon contributes to the remarkable venom phenotypic variability found across the palm-pitviper phylogeny. A possible mechanism for the occurrence of type-I/type-II venom phenotypic dichotomy is discussed.

## Graphical abstract

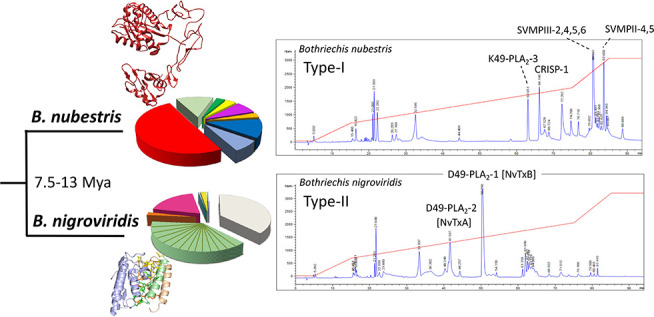

## 1 Introduction

Animal venoms are integrated phenotypes and intrinsically ecological traits evolved independently in a broad phylogenetic range of organisms across all major phyla of the animal kingdom under selective pressure for subjugating prey, deterring competitors or defending themselves from predators.^1,2^ The emergence of venom at the base of the caenophidian radiation in the wake of the Cretaceous–Paleogene mass extinction, approximately 60–50 million years ago,^3–5^ enabled small ancestral snakes to radiate into novel ecosystems and compete for the resources. Although a comprehensive understanding of the molecular processes that generated such biodiversity remains largely elusive, venoms of extant snakes are thought to have been shaped by complex intrinsic and extrinsic factors, such as ontogeny, feeding ecology, predator–prey coevolutionary “arms race,” inter- and intraspecific competition, and geographic distribution and population ecology.^2,6^ In extant snakes, venom plays important ecological roles that predominantly involve predation and/or defense.^7–9^ Thus, the evolution of the continuous spectrum of venom phenotypes to their present-day variability landscape should be understood on a macroevolutionary scale in the context of the natural history of the organisms that produce them.^5,10–12^ In this sense, mapping the venom phenotypic profiles of a species clade across its phylogeny and geographic range can lend a great deal of insight for understanding the ecological and evolutionary dynamics of venom variation.^7,9,13–25^

*Bothriechis* (Serpentes: Viperidae) represents a monophyletic basal genus of 11 recognized species (*B. supraciliaris*, *B. schlegelii*, *B. nigroviridis*, *B. nubestris*, *B. lateralis*, *B. guifarroi*, *B. thalassinus*, *B. marchi*, *B. bicolor*, *B. aurifer*, *B. rowley*)^26^ and another 8 proposed taxa within the *B. schlegelii* species group, including 4 revalidated (*B. nigroadspersus*, *B. torvus*, *B. khwargi* and *B. nitidus*) and another 4 proposed (*B. rasikusumorum* sp. nov, *B. klebbai* sp. nov, *B. rahimi* sp. nov, and *B. hussaini* sp. nov) new species.^27^ However, Reyes-Velasco has revised the proposed taxonomic changes and concluded that “only *B. nigroadspersus* should be maintained as valid,” while most taxa proposed by Arteaga et al.^27^ should be considered geographic synonyms, or at most, as subspecies of *B. schlegelii*.^28^

*Bothriechis* has a most recent common ancestor with *Ophryacus* in Middle America approximately 14–18 million years ago (Mya).^29–31^ Its current phylogeny, biogeographic distribution and intrageneric venom variability have been shaped in the course of a complex evolutionary history over a period of intense geologic upheaval in Middle America during late Tertiary time that fragmented *Ophryacus* to the northwest and *Bothriechis* to the southwest, where subsequent orogenic activity of the proto-Antillean island arc marked the beginning of the intrincate history of vicariant fragmentation and speciation within genus *Bothriechis*.^32^

Extant *Bothriechis* taxa are relatively slender to medially robust arboreal, prehensive-tailed rattleless pitvipers, commonly called palm-pitvipers. However, *B. supraciliaris* tends to spend more time on the ground than other palm-pitvipers.^33^ Species found below 1000 m altitude tend to have nocturnal prey habits while species that occupy habitats above 1500 m altitude tend to predate during the day light.^34^ As is typical for arboreal ambush predators, palm-pitvipers seize and hold the prey until it has succumbed to the venom.^34^ Although detailed dietary studies for most *Bothriechis* species are scarce, most species are known to be generalists which hunt arboreal prey such as anoles, small birds, and occasionally bats.^34,35^ On the other hand, the stomach contents of four dissected specimens of *B. supraciliaris* consisted mostly of small rodents of the forest floor.^33^

Except for the widespread palm-pitviper species complex previously subsumed under *B. schlegelii* (*sensu lato*), which inhabit humid lowlands and foothills from southern Mexico southeastward on the Atlantic plains and lowlands through Central America to northern South America in Colombia and Venezuela, and mesic forest at elevations almost from sea level to 2,640 m on the Pacific versant and lowlands of Costa Rica, Panama, Colombia, Ecuador, and Peru,^27^ all the other palm-pitvipers are confined to disjunt “sky-islands” between the Isthmus of Tehuantepec in southern Mexico and Central Panamá.^34,36^

Extensive compositional venomics profiling across *Bothriechis* has been conducted for 8 of the 11 recognized species, namely *B. lateralis* (side-striped palm pit viper) and *B. schlegelii* (eyelash pit viper) from Costa Rica;^37,38^ *B. nigroviridis* (black-speckled palm pitviper) from Costa Rica;^39,40^ *B. supraciliaris* (blotched palm-pit viper) from Costa Rica;^41^ *B. thalassinus* (Merendon palm-pitviper), *B. bicolor* (Guatemalan palm-pitviper), and *B. aurifer* (yellow-blotched palm-pitviper) from Guatemala, and *B. marchi* (Honduran palm-pitviper) from Honduras.^18^ The remarkable degree of venom phenotypic variability found across palm-pitviper phylogeny, which is reflected in notable differential activity within the coagulotoxic and postsynaptic neurotoxic activity of Bothriechis venoms,^42^ makes this clade represent an opportunity to infer key nodes of adaptive venom pattern variation underlying the insular biogeographic isolation across Middle American palm-pitvipers. Here we report the compositional and functional profile of the venom of the Talamancan palm-pitviper (*B. nubestris*) endemic to Costa Rica.^26^ This species is found in north and central parts of the Cordillera de Talamanca to the provinces of San José, Cartago, and Limón at altitudes between 2100 m above sea level on Cerro de la Muerte to close to 3000 m asl in San Gerardo de Dota, in the transition area between the premontane rainforest and the montane rainforest.

Comparison of their venom proteomes and published venom gland transcriptomes indicates that *B. nubestris* and its closest relative, *B. nigroviridis*, possess highly conserved venom gland transcriptomes, which appear to undergo post-transcriptionally regulated translation into divergent (type-I vs type-II, respectively) venom phenotypes. A possible mechanism for this venom phenotypic dichotomy is discussed.

## 2 Materials and Methods

### 2.1 Venoms and Antivenom

Venom from *B. nubestris* was obtained from one specimen collected in San Gerardo de Dota, San José, Costa Rica (LIAP 0292, latitude 9.55014, longitude −83.807450, 2200 m above sea level). After collection, venom was centrifuged to remove debris, lyophilized and kept at −20 °C until use. Venom from *Bothrops asper* from a pool of at least 20 specimens from the Pacific versant of Costa Rica was used for comparative purposes. To compare the RP-HPLC profiles of the venom proteomes of *B. nubestris* and *B. nigroviridis*, venom from a specimen of the latter species maintained at the Laboratory for the Investigation of Dangerous Animals (LIAP, Instituto Clodomiro Picado, Costa Rica) was employed. For neutralization assays, the polyvalent antivenom manufactured at Instituto Clodomiro Picado (PoliVal-ICP, Batch 5880417; 51.4 mg whole IgG/mL; expiry date: 04/2020) was used.

### 2.2 Decomplexation of the Venom Proteome by Reverse-Phase HPLC and SDS-PAGE

Reverse-phase (RP) HPLC fractionation of *B. nubestris* venom was performed on an Agilent 1200 chromatograph. Crude venom samples (2 mg) were dissolved in 200 μL of solution A (0.1% trifluoroacetic acid; TFA), centrifuged at 15,000*g* for 5 min, and separated on a C_18_ (250 × 4.6 mm, 5 μm particle size; Teknokroma) column. Elution was monitored at 215 nm, at a flow rate of 1 mL/min, applying a gradient of 0.1% TFA in Milli-Q water (solution A) and acetonitrile (solution B) as follows: 0% B for 5 min, 0–15% B over 10 min, 15–45% B over 60 min, 45–70% B over 10 min, and 70% B over 9 min.^43^ Fractions were collected manually, dried in a vacuum centrifuge (SpeedVac, Thermo Savant), and submitted to SDS-PAGE protein separation in precast 12% polyacrilamide gels (Bio-Rad) under reducing and nonreducing conditions. Protein bands were stained with Coomassie blue R-250. The relative abundance of the venom components was estimated applying the hierarchical three-level quantification protocol described by Eichberg et al.:^44^ in step 1, the reverse-phase column eluate was monitored at the absorbance wavelength of the peptide bond to estimate the relative abundances of the chromatographically separated fractions by peak area integration. The relative abundances of proteins coeluting in the same reverse-phase fraction were estimated in the second level of quantification by densitometry of Coomassie-stained SDS polyacrylamide gels. In the third level of quantification, the relative abundances of different proteins comigrating in the same SDS-PAGE band were estimated based on the relative ion intensities of the peptide ions assigned by MS/MS analysis to each of the comigrating proteins.

### 2.3 Venom Peptidome

RP-HPLC fractions that did not show any protein band were dried in a vacuum centrifuge (SpeedVac, ThermoSavant), redissolved in 15 μL of water containing 0.1% formic acid, and submitted to LC-MS/MS. Peptides were separated by nano-Acquity Ultra Performance LC (UPLC) using a BEH130 C_18_ (100 μm × 100 mm, 1.7 μm particle size) column in-line with a Waters SYNAPT G2 High Definition Mass Spectrometry System. The flow rate was set to 0.6 μL/min, and the column developed a linear gradient of 0.1% formic acid in Milli-Q water (solution A) and ACN (solution B) with the following conditions: isocratically 1% B for 1 min, followed by 1–12% B for 1 min, 12–40% B for 15 min, 40–85% B for 2 min. For peptide ion fragmentation by collision-induced dissociation tandem mass spectrometry (CID-MS/MS), the electrospray ionization (ESI) source was operated in positive ion mode, and both singly and multiply charged ions were selected for CID-MS/MS at sample cone voltage of 28 V and source temperature of 100 °C. The UPLC eluate was continuously scanned from 300 to 1990 *m*/*z* in 1 s, and peptide ion MS/MS analysis was performed over the range *m*/*z* 50–2000 with a scan time of 0.6 s. MS/MS fragmentation spectra were interpreted manually (*de novo* sequencing). Amino acid sequence similarity searches were performed at https://blast.ncbi.nlm.nih.gov/Blast.cgi against the nonredundant protein sequences database, using the default parameters of the BLASTP program (Altschul et al., 1990).^45^

### 2.4 Mass Spectrometry

#### 2.4.1 Electrospray Ionization Mass Determination of *B. nubestris* Venom Proteins

RP-HPLC fractions were dissolved in 50% acetonitrile and 0.1% formic acid and analyzed by direct infusion (flow rate 5 μL/min) using a Q-Exactive Plus mass spectrometer (Thermo) with a heated electrospray ionization (HESI) ion source to determine the mass of intact RP-HPLC separated *B. nubestris* venom proteins. MS spectra were acquired in positive mode, using 3.9 kV spray voltage, full MS scan range from 800 to 2500 *m*/*z*, 140000 resolution, and an AGC target of 3 × 10^6^). Monoisotopic and isotope-averaged molecular masses were calculated by deconvolution of the isotope-resolved multiply charged MS1 mass spectra.

#### 2.4.2 Bottom-Up Venomics

SDS-PAGE bands of the RP-HPLC fractionation of *B. nubestris* venom were excised from Coomassie-stained gels and submitted to bottom-up venomics analysis.^44,46^ To this end, the gel plugs were subjected to automated disulfide bond reduction (10 mM dithiothreitol, 20 min at 60 °C) and cysteine alkylation (50 mM iodoacetamide, 15 min in the dark at room temperature), followed by digestion with sequencing-grade trypsin, using an Intavis workstation. The resulting tryptic peptides were submitted to nESI-MS/MS on a Q-Exactive Plus mass spectrometer (Thermo). Twelve μL of each tryptic digest was separated at 200 nL/min with a 3 μm particle, 15 cm × 75 μm C_18_ Easy-spray analytical column using a nano-Easy 1200 chromatograph (Thermo). A gradient from 0.1% formic acid (solution A) to 80% acetonitrile with 0.1% formic acid (solution B) was developed: 1–5% B in 1 min, 5–26% B in 25 min, 26–79% B in 4 min, 79–99% B in 1 min, and 99% B in 4 min, for a total time of 35 min. MS spectra were acquired in positive mode at 1.9 kV, with a capillary temperature of 200 °C, using 1 scan at 400–1600 *m*/*z*, maximum injection time of 100 ms, AGC target of 3 × 10^6^, and orbitrap resolution of 70,000. The top 10 ions with 2–5 positive charges were fragmented with AGC target of 1 × 10^5^, maximum injection time of 110 ms, resolution of 17,500, loop count of 10, isolation window of 1.4 *m*/*z*, and a dynamic exclusion time of 5 s. MS/MS spectra were processed for the assignment of peptide matches to known protein families by similarity with sequences contained in the venom gland transcriptomic database of *B. nubestris* and *B. nigroviridis*,^31^ using Peaks X (Bioinformatics Solutions). Cysteine carbamidomethylation was set as a fixed modification, while deamidation of asparagine or glutamine and methionine oxidation were set as variable modifications, allowing up to 3 missed cleavages by trypsin. Parameters for match acceptance were set to FDR ≤ 0.1%, −10lgP protein score ≥ 150, unique peptides ≥ 1, and significant peptides. Proteins matched with a less stringent parameter (i.e., protein score of ≥ 140) were accepted after manual verification of their corresponding peptide spectra. Finally, the relative abundance of each protein family (% of total venom proteins) was estimated by integration of the RP-HPLC chromatogram at 215 nm, using Agilent’s Chem Station B.04.01 software. For peaks that presented more than one SDS-PAGE band, percent distribution was assigned by densitometry, using a ChemiDoc recorder and Image Lab v.2.0 software (Bio-Rad). When detection of several protein types in the same SDS-PAGE band digest occurred, their percent distribution was estimated based on integrating the peptide feature area under the curve from the nLC-MS/MS run using Peaks X (Bioinformatics Solutions). Recording the eluate at the absorbance wavelength of the peptide bond [190–230 nm], and applying the Lambert–Beer law (A = εcl, where A = absorbance; ε is the molar absorption [extinction] coefficient, [M^–1^ cm^–1^]; c = concentration [M]; and l = light path length [cm]), the percentage of total peptide bond concentration represents a good estimate for a toxin’s % by weight (g toxin/100 g venom proteome).^47,48^

### 2.5 Determination of *In Vitro* Venom Activities

#### 2.5.1 Phospholipase A_2_ Activity

Different amounts (2.5, 5, 10, 20, and 40 μg) of venom from *B. nubestris* or *B. asper* in 25 μL of water were added to 200 μL of 10 mM Tris–HCl, 10 mM CaCl_2_, 0.1 M NaCl, pH 8.0 buffer, in microplate wells. Twenty-five μL (1 mg/mL in acetonitrile) of the monodisperse synthetic substrate 4-nitro-3-octanoyloxy-benzoic acid (NOBA) were then added.^49^ After the reaction mixtures were incubated for 60 min at 37 °C, PLA_2_ activity was determined by quantifying the release of the cromophore 4-nitro-3-hydroxybenzoic acid at 405 nm using a microplate reader (Thermo). One unit of PLA_2_ activity was defined as the change of 1 absorbance unit. Samples where venom was omitted were included as a blank control. All assays were performed in triplicates.

#### 2.5.2 Proteolytic Activity on Azocasein

Variable amounts of *B. nubestris* or *B. asper* venoms (1.25, 2.5, 5, 10, 20, and 40 μg dissolved in 20 μL of 50 mM Tris–HCl, 0.15 M NaCl, 5 mM CaCl_2_, pH 8 buffer) were added to 100 μL of azocasein (10 mg/mL, dissolved in the same buffer). The mixtures were incubated for 90 min at 37 °C, and the reaction was stopped by adding 200 μL of 5% trichloroacetic acid. After centrifugation at 1500 rpm for 5 min, 150 μL of each supernatant was transferred to 96-well microplates, mixed with 150 μL of 0.5 M NaOH, and absorbance was recorded at 450 nm^50^ using a Multiskan reader (Thermo). The activity was expressed as the increase in absorbance at 450 nm in comparison to the substrate alone. Assays were performed in triplicate wells.

#### 2.5.3 Gelatin Zymography

Zymography to determine venom gelatinolytic activity was carried out under unreduced conditions. The venom (10 μg) was subjected to SDS-PAGE on 12% gels containing type A gelatin (Sigma Chemical Co.) at a 0.25 mg/mL concentration. After washing for 1 h with 1% Triton X-100 to remove SDS, the gels were incubated at 37 °C for 24 h in 50 mM Tris–HCl buffer, pH 8.0, containing 5 mM CaCl_2_. Gels were stained with Coomassie Blue R-250.

#### 2.5.4 Coagulant Activity

0.2 mL of citrated human plasma was incubated for 5 min at 37 °C. Afterward, 100 μL of solutions containing 20 μg of *B. nubestris* venom, 20 μg of *B. asper* venom (positive control), or only phosphate-buffered saline (PBS; 0.12 M NaCl, 40 mM sodium phosphate, pH 7.2, negative control) was added followed by incubation at 37 °C, and the clotting times were recorded. Assays were performed in triplicates.

### 2.6 *In Vivo* Venom Activities

#### 2.6.1 Animal Ethics

All procedures involving animals were approved by the Institutional Committee for the Use and Care of Animals (CICUA) of the University of Costa Rica (permission No. 052-2019).

#### 2.6.2 Myotoxic Activity and Effect on Hematocrit

Groups of five mice (18–20 g) received an intramuscular injection of 50 μg of either *B. nubestris* or *B. asper* venom in 50 μL of PBS in the right gastrocnemius muscle. The control group received 50 μL of PBS. Three hours later blood samples were taken from the tail of each mouse into heparinized capillary tubes and centrifuged to obtain plasma and for hematocrit measurement. Four μL of plasma were used to determine the creatine kinase (CK) activity using an UV kinetic assay (Wiener Lab, Germany). CK activity was expressed in units/mL.

#### 2.6.3 Venom Lethality

Doses of 5, 10, 20, 30, and 40 μg of *B. nubestris* venom, dissolved in 200 μL of PBS, were injected in the caudal vein in groups of five CD-1 mice (16–18 g body weight). Deaths were recorded after a period of 24 h. The median lethal dose (LD_50_) was calculated by Probit analysis^51^ using BioStat software v.5.2.5 (Analysoft).

#### 2.6.4 Hemorrhagic Activity

Groups of three CD-1 mice (18–20 g) were injected intradermally in the ventral abdominal region with various doses of *B. nubestris venom* (1.25, 2.5, 5, 10, and 20 μg) in 100 μL of PBS. Two hours postinjection mice were sacrificed by CO_2_ inhalation and their skins removed. The hemorrhagic areas on the inner side of the skin were measured. Hemorrhagic activity was expressed as the Minimum Hemorrhagic Dose (MHD), corresponding to the dose of venom that induces a hemorrhagic area of 10 mm diameter.^52^

#### 2.6.5 Antivenom Neutralization of Venom Lethality and Hemorrhagic Activity

Venom of *B. nubestris* was incubated for 30 min at 37 °C with PoliVal-ICP antivenom at ratios of 0.5, 1, 2, and 4 mg of venom/mL antivenom, (corresponding to 9.7, 19.4, 38.9, and 77.8 mg venom/g antivenom). Venom incubated with PBS in the same conditions was used as a control. Groups of five CD-1 mice (16–18 g) received intravenous injections of 200 μL of the preincubated solutions, which contained a fixed challenge dose of 4 LD_50_s (86 μg) of venom and increasing amounts of PoliVal-ICP antivenom. Deaths were recorded after 24 h and the effective dose 50% (ED_50_) was determined by Probit analysis^51^ using BioStat software v.5.2.5 (Analysoft). The potency (i.e., the amount of venom (mg) neutralized per 1 mL of antivenom, was calculated as P = [(n-1)/ED_50_] × LD_50_, where “n” is the number of LD_50_s used as challenge dose to determine the ED_50_. “n-1” is used because at the end point of the neutralization assay the activity of one LD_50_ remains unneutralized causing the death of 50% of mice.^53,54^

Samples containing 18.5 μg of *B. nubestris* venom were incubated for 30 min at 37 °C with PoliVal-ICP antivenom (at 0.5 and 2 mg venom/mL antivenom ratios, corresponding to 9.7 and 38.9 mg venom/g antivenom, respectively). Venom incubated with PBS in the same conditions was used as a control. Groups of four CD-1 mice (18–20 g) were injected intradermally in the ventral abdominal region with 100 μL of the preincubated venom:antivenom solutions. The hemorrhagic activity was determined as described above.

### 2.7 Statistical Analyses

The significance of differences between means of two groups was assessed by Student’s *t*-test. ANOVA with posthoc Tukey HSD was used for comparing the means of three groups. Differences with *p* < 0.05 were considered significant.

### 2.8 Data Availability

Mass spectrometry data have been deposited with the MassIVE repository under accession number MSV000096598 (ftp://massive.ucsd.edu/MSV000096598/) and in ProteomeXchange with accession number PXD058628.

## 3 Results and Discussion

### 3.1 The Dominating SVMP Proteome and the Venom Peptidome of the Talamancan Palm-Pitviper *B. nubestris* [Fn fn1]

The venom proteins of *B. nubestris* were separated into 24 RP-HPLC fractions (Figure 1A), which were analyzed on SDS-PAGE gels under reduced (Figure 1B) and nonreduced (Figure 1C) conditions.

**Figure 1:**
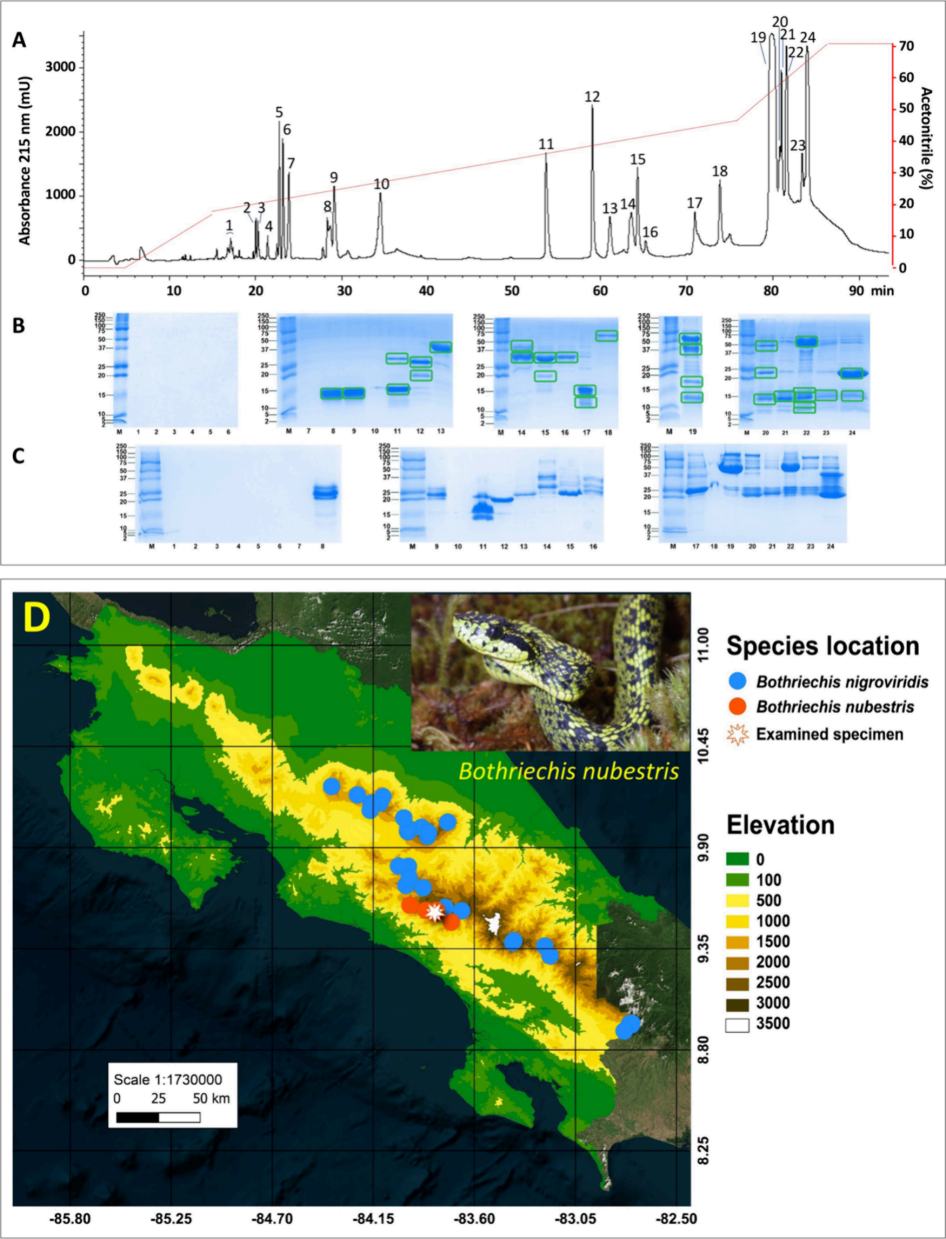
Pre-MS decomplexation of the Talamancan palm-pitviper, *Bothriechis nubestris*, venom proteome. Panel A, Reverse-phase fractionation of 2 mg of crude venom. Chromatographic fractions were manually collected and analyzed by SDS-PAGE under reduced (panel B) and nonreduced (panel C) conditions. Coomassie blue-stained bands framed with green rectangles were excised from the gels and used for downstream bottom-up proteomic analyses. Panel D displays the geographical distribution of *B. nubestris* and, for comparison, that of *B. nigroviridis* and highlights the collection site (latitude 9.55014, longitude −83.807450, 2200 m above sea level) of the venom of the donor specimen LIAP 0292 used in this study.

Quantitative bottom-up proteomic analysis of the Coomassie blue-stained SDS-PAGE protein bands was applied to uncover the identity and relative abundance of the *B. nubestris* venom peptidome and proteome. The peptides and parent proteins identified matching the MS/MS spectra against the *B. nubestris* and *B. nigroviridis* transcriptomic databases are displayed in , and their quantification is shown in . Out of 41 toxin-coding transcripts that make up the venom gland transcriptome of *B. nubestris*,^31^ 26 unique transcripts were found unevenly translated into its venom arsenal. These data are integrated into the pie chart shown in Figure 2, where the venom composition is broken down into toxin classes, and the specific transcripts translated into the venom proteome are highlighted.

**Figure 2:**
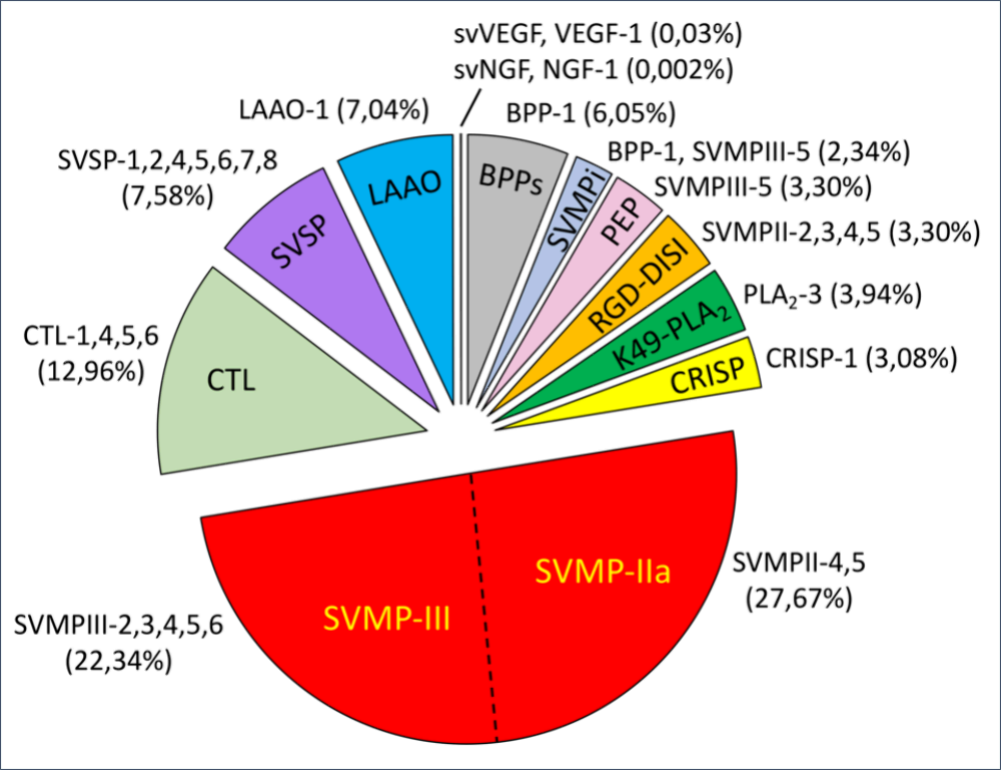
Overview of the venom composition of *Bothriechis nubestris*, venom peptidome, and proteome. The pie chart displays the compilation of the bottom-up and transcriptome-aided mass spectrometric outcomes. Highlighted are the relative abundances of the 10 venom toxin classes (expressed as % by weight of the total of venom proteome) and the identity of the individual components named by the acronym of the 26 coding transcripts that contribute to the venom with primary or proteolytically derived components. Note that the endogenous tripeptide inhibitors of metalloproteases (SVMPi) and the SVMPIII-5 proteolytic peptides (PEP) are not included in the counting of toxin classes. Acronym keys: SVMP-IIa and SVMP-III, single metalloproteinase derived from a SVMP-II, and snake venom metalloproteinase of class PIII class, respectively; CTL, C-type lectin-like; SVSP, snake venom serine proteinase; LAAO, L-amino acid oxidase; svVEGF and svNGF, snake venom vascular endothelial growth factor and snake venom nerve growth factor, respectively; BPP, bradykinin-potentiating peptide; PEP, proteolytic peptide; RGD-DISI, disintegrin bearing the arginine-glycine-aspartic acid integrin-binding tripeptide motif; K49-PLA_2_, Lysine 49 phospholipase A_2_-like proteins; CRISP, cysteine-rich secretory protein.

Talamancan palm-pitviper venom is dominated by snake venom metalloproteinases, PIIa-SVMP molecules (Fox and Serrano (2008) nomenclature), which account for 27.7% of the venom proteome, and SVMP-III 2,3,4,5,6, which together account for 22.3% of the toxin arsenal of *B. nubestris*. No transcript encoding a PI-SVMP domain alone was found in the *B. nubestris* or *B. nigroviridis* transcriptomic databases. Conversely, the two PIIa-SVMP molecules identified in RP-HPLC fraction 24 (Figure 1) are proteolytically derived from PII-SVMPs 4 and 5 precursors.^55^ In this process, PII-SVMPs 4 and 5 released their medium-size RGD-disintegrin domains into the venom.^56,57^ Two other PII-SVMPs (encoded in transcripts Bnubes-SVMPII-2 and 3) also released their medium-sized RGD-disintegrin domains into the venom arsenal. However, lack of proteomic evidence for intact PIIa domains derived from these PII-SVMP precursors supports common observations that certain metalloproteinase domains, particularly from PII precursors, become degraded during their post-translational proteolytic processing.^58^ Our results show that a large part of the venom peptidome found in *B. nubestris* venom was generated by degradation of the metalloprotease domains of SVMP-II 2, 3, 4, and 5 and SVMP-III 2, 3, and 5 (). Although the structural basis of the destabilization of PI domains of SVMP-III and the PIIa domains of PII-SVMPs, as well as the underlying mechanism of degradation and the biological significance of their presence in venom, are elusive, pyroglutamyl tripeptides ZKW and ZNW found in the SVMP-derived *B. nubestris* peptidome () have been reported in many snake venoms^16,59,60^ and characterized as weak endogenous inhibitors (IC_50_ in the range of 0.15–0.95 mM) of the fibrinogenolytic activity of multiple snake venom Zn^2+^-SVMPs.^61–63^ These peptide inhibitors regulate the proteolytic activities of SVMPs in a reversible manner under physiological conditions.^62^ It is thought that pyroglutamyl tripeptides may protect glandular tissues and venom factors from the proteolytic activity of SVMPs stored at a high concentration in an inactive but competent state for many months in the lumen of the venom gland. The role of tetrapeptide ZTNW derived from degradation of SVMPIII-2 and 3, and/or SVMPII-3 and 5 () requires further research.

Another transcript contributing to the *B. nubestris* venom peptidome is Bnubes-BPP-1. This single-copy gene transcript encodes the common precursor for endogenous SVMP tripeptide inhibitors (SVMP_i_s) and bradykinin-potentiating peptides (BPPs). SVMP_i_ ZHW eluted in RP-HPLC fraction 5 (Figure 1, ) and the BPP ZHWSPGHHIPP, eluted in RP-HPLC fraction 10 (Figure 1, ) and accounting 3.45% of the venom proteome, are released into the venom proteome by the proteolytic processing of their common precursor.^64,65^ BPPs are inhibitors of the angiotensin I-converting enzyme, which enhance the hypotensive effect of circulating bradykinin, thus contributing to cardiovascular shock of the snake’s envenomed organism.^66–68^ Putative BPPs represent 6% of the *B. nubestris* venom (Figure 2, ).

### 3.2 The Medium to Low Abundance *Bothriechis nubestris* Venom Proteome

In descending order of abundance, the ranking of the medium to low abundance venom toxin classes is (Figure 2): C-type lectin-like (CTLs 1,4,5,6, 12.96% of the venom proteome); Snake venom serine proteinases (SVSPs 1,2,4,5,6,7,8, 7.58%); L-amino acid oxidase (LAAO-1, 7.04%); Phospholipase A_2_ (PLA_2_-3, 3.94%); PII-Disintegrins (SVMPII-2,3,4,5, 3.77%); Cysteine-rich secretory protein CRISP-1 (3.08%); Snake venom vascular endothelial growth factor (VEGF-1, 0.03%); and Snake venom nerve growth factor, svNGF-1 (0.002%). Comparison of SDS-PAGE analysis of the RP-HPLC fractions containing CTL proteins (Figure 1B, HPLC fractions 17, 20, 21, 22, and 23) clearly indicated that these toxins are canonical dimeric molecules apparent nonreduced and reduced molecular mass of 25–30 kDa and 14–16 kDa, respectively).^69^ BLAST analysis showed that Bnubes-CTL-4 displays extensive amino acid sequence homology (69% identity; 81% similarity) to α-type subunits from a number of both Asiatic (Gloydius, Protobothrops, and Trimeresurus) and Nearctic (Crotalus) snake species. On the other hand, Bnubes-CTLs 1, 5, and 6 showed 88–92% identity/94–95% similarity to β-type subunits of CTLs from the same Old and New World genera. CTLs 4 and 5 were the only CTLs identified in RP-HPLC fractions 17B and 22, strongly suggesting that these molecules form a canonical αβ heterodimeric association. The coelution of more than two CTL subunits in the same protein band (e.g., CTLs 4, 5, and 6 in fraction 21, 22B, and all four CTLs in fraction 17A and 20C) might suggest the occurrence of different αβ heterodimers sharing the CTL-4 α subunit and/or different homodimeric arrangements.

### 3.3 *In Vitro* and *In Vivo* Biological Activities of *B. nubestris* Venom and Neutralization by PoliVal-ICP Antivenom

The toxin composition of *B. nubestris* venom, characterized by the predominance of putative hemorrhagic PIII-SVMPs and RGD-disintegrin molecules; fibrinogenolytic PIIa-SVMPs; vasoactive BPPs; myotoxic K49-PLA_2_; and blood coagulotoxic SVSPs, LAAO and CTLs, could suggest a hemotoxic and cytotoxic venom.^70–84^ However, the absolute literature silence on toxicity on natural prey or human envenomings precludes a reasonable knowledge-based guess of the biological activities of *B. nubestris* venom. Here, we report functional *in vitro* assays to assess phospholipase A_2_, proteolytic (caseinolytic), collagenolytic (gelatin zymography) and coagulant (clotting time) activities; *in vivo* lethality, myotoxicity and hemorrhagic activities; and the neutralization of hemorrhagic and lethal activities by the polyvalent antivenom manufactured at Instituto Clodomiro Picado (PoliVal-ICP).

Consistent with the lack of D49 PLA_2_s in the proteomic analysis, *in vitro* assays showed that the venom of *B. nubestris*, at the highest amount (40 μg) tested, had no PLA_2_ activity (Figure 3A). On the other hand, *B. nubestris* venom displayed notable proteolytic activity on azocasein (Figure 3B, left panel), slightly higher than that recorded for the positive control, *B. asper* venom, and on gelatin zymography (Figure 3B, right). *B. nubestris* venom (20 μg) and the negative control (PBS) did not cause clotting of citrated human plasma after 60 min, while the clotting time recorded for the same amount of *B. asper* venom was 17.3 ± 2.1 s.

**Figure 3:**
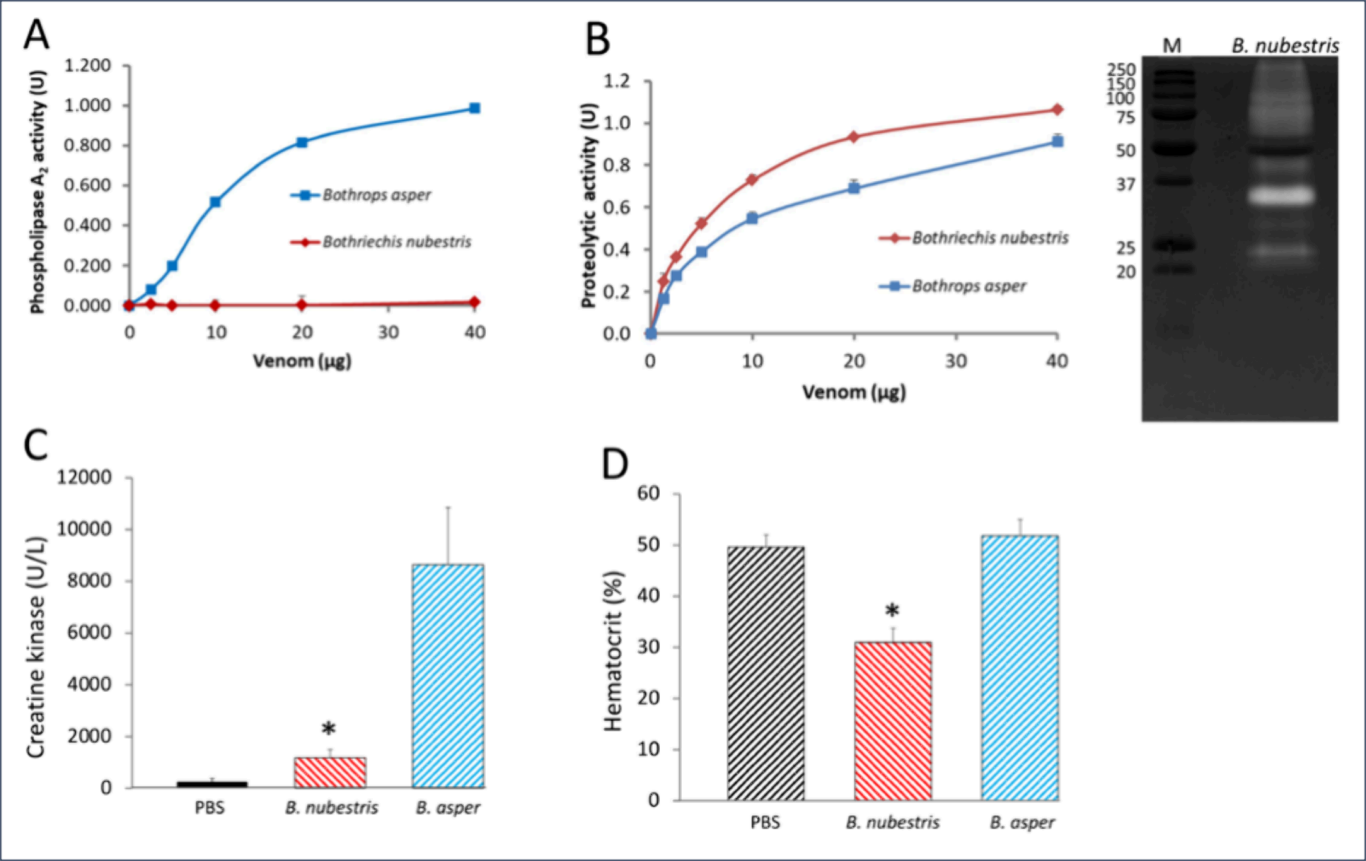
*In vitro* and *in vivo* determination of the biological activities of *B. nubestris* venom. Panel A, Concentration-dependent assay of the phospholipasic activity of *B. nubestris* venom. The effect of different amounts of *B. nubestris* venom or *B. asper* venom (used as positive control) to release the cromophore 4-nitro-3-hydroxybenzoic acid from the monodisperse synthetic chromogenic substrate 4-nitro-3-octanoyloxy-benzoic acid was taken as a proxy for phospholipase activity of the venom. Panel B, Left display, concentration-dependent measurement of the proteolytic activity of *B. nubestris* venom on azocasein. For comparison, the assay was performed in parallel using the well-studied venom of *Bothrops asper*. Right display: visualization of collagenolytic activity by gelatin zymography. Panel C and D, determination of myotoxic activity and effect on hematocrit, respectively, of the intramuscular injection of 50 μg of *B. nubestris* or *B. asper* (control) venoms in the right gastrocnemius muscle of mice. * stands for statistically significant (*p* < 0.01) effect of *B. nubestris* venom compared to PBS and *B. asper* venom.

*In vivo* experiments showed a minor, although statistically significant (*p* < 0.01), increase of the creatine kinase (CK) activity in the plasma of mice injected with 50 μg of *B. nubestris* venom in the right gastrocnemius muscle compared to mice injected with PBS alone (Figure 3C), which may be attributed to its low (4%) proportion of a K49 PLA_2_ homologue (Figure 2). Conversely, the same amount of *B. asper* venom used as positive control, caused a pronounced increase of CK plasmatic activity (Figure 3C). Mice injected intramuscularly with *B. nubestris* venom presented a significant decrease in blood hematocrit (31 ± 3%, Figure 3D) 3 h after injection, compared with control mice injected with PBS alone (50 ± 2%). Difference in hematocrit values for PBS versus *B. nubestris* venom, as well as *B. asper* versus *B. nubestris* venom, are statistically significant (*p* < 0.01). Such drop in hematocrit is likely to be a consequence of the hemorrhagic activity of the venom.

In mice (18–20 g body weight) *B. nubestris* venom exhibited a minimum hemorrhagic dose (MHD) of 1.85 μg. The hemorrhagic activity of 10 MHD of *B. nubestris* venom was also neutralized by PoliVal-ICP antivenom: while 18.5 μg of venom induced a hemorrhage of 397 ± 60 mm^2^, 500 μL (25.7 mg) antivenom/mg venom caused significant (*p* < 0.01) reduction of the area of hemorrhage to 216 ± 64 mm^2^ and a further decrease to 30 ± 17 mm^2^ was achieved when 2000 μL (102.2 mg) antivenom/mg venom was used. This corresponds to 0.5 mg of venom/mL antivenom or 9.8 mg of venom/g antivenom.

*Bothriechis nubestris* venom exhibited an intravenous (i.v.) median lethal dose (LD_50_) of 21.5 (95% confidence interval: 15.6–29.5) μg/mouse, which corresponds to 1.26 (0.92–1.74) μg/g mouse (17 g body weight). The lethality of 4 i.v. LD_50_s of *B. nubestris* venom was neutralized by the polyvalent ICP (PoliVal-ICP) antivenom with a potency (P) of 0.5 mg of venom/mL of antivenom (P = 9.7 mg V/g AV). The murine LD_50_ of *B. nubestris* corresponds to an allometric translated human LD_50_ of (murine LD_50_ × 3)/37 = 0.102 (0.075–0.141) μg/g human body,^85^ suggesting that 5.25–9.87 mg could cause the death of a 70 kg adult human. Although there is no case history of envenoming by *B. nubestris*, in the event of a bite, treatment with PolyVal AV seems to be a good option: for an estimate of 5–20 mg venom yield, 1–4 vials would be needed to neutralize the venom deployed in the bite.

### 3.4 Comparison of Venom Gland Transcriptomes and Venom Proteomes Provides Clues on the Origin of the Venom Phenotypic Dichotomy between *B. nubestris* and *B. nigroviridis*

Database searches of the MS/MS fragmentation spectra of the tryptic digests of the RP-HPLC fractions matched, in most chromatographic peaks, orthologous hits in both the *B. nubestris* and the *B. nigroviridis* venom gland transcriptomes (). A notable exception is Bnubes-PLA2-3, a K49-PLA_2_ exclusively found in the *B. nubestris* transcriptome and proteome. Transcripts Bnubes-PLA2-1 and Bnubes-PLA2-2 are highly conserved or identical to Bnigro-PLA2-1 and Bnigro-PLA2-2, respectively. Both encode D49-PLA_2_ precursor molecules, which share >80% sequence identity with, respectively, the basic (B) and the acidic (A) subunits of heterodimeric beta-neurotoxic PLA_2_ molecules distributed in snake venoms within genera *Gloydius*,^86^ *Sistrurus*,^14^ *Crotalus*,^13,87,88^ *Bothriechis*,^39^ *Ophryacus*,^89^ and *Mixcoatlus* ^90,91^ (Figure 4).

**Figure 4:**
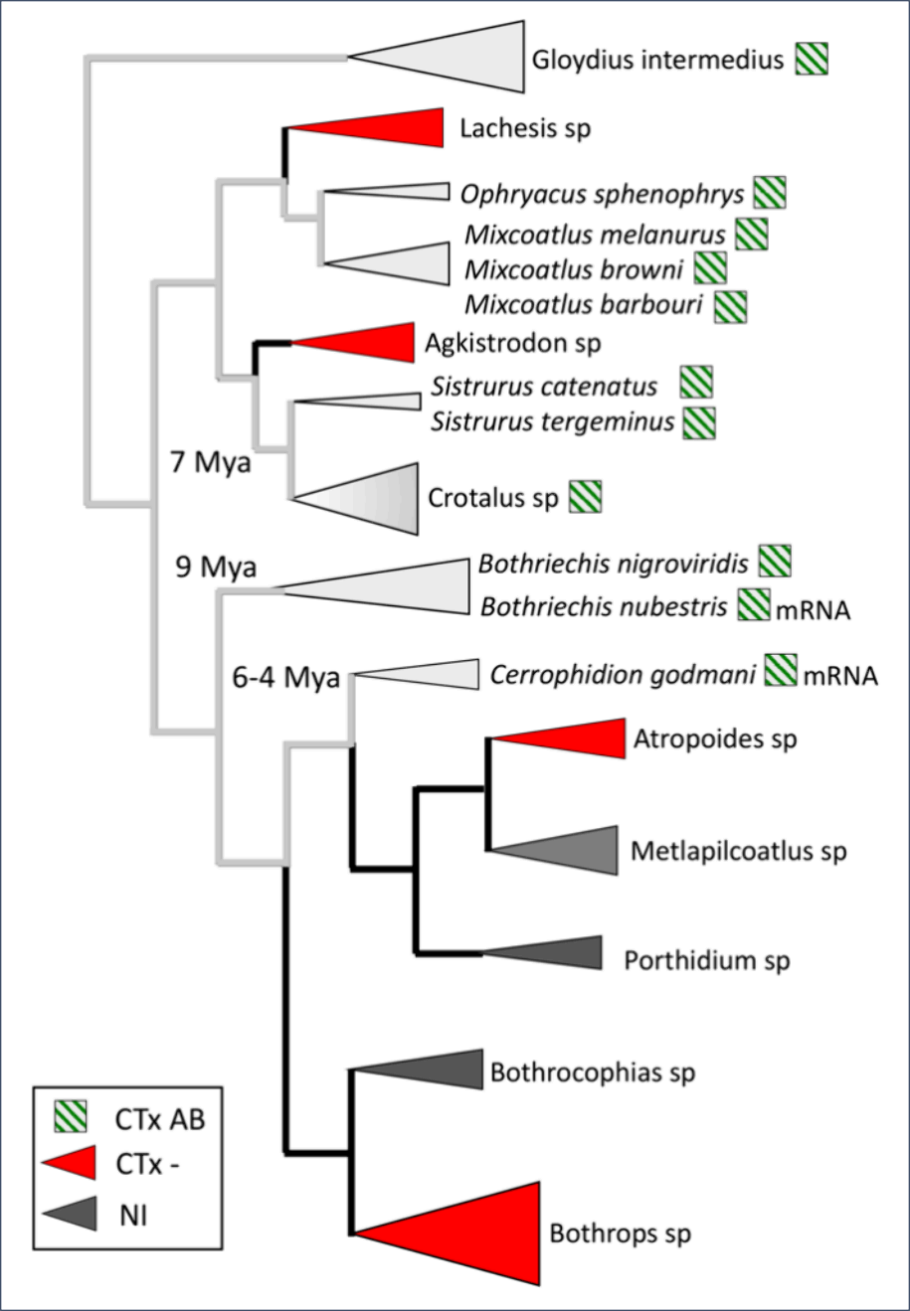
Distribution of crotoxin-like heterodimeric PLA_2_s. Scheme of the cladogenesis of Crotalinae in the American continent highlighting taxa in which the occurrence of crotoxin-like heterodimeric PLA_2_ molecules have been described in their venom (squares with green oblique lines) or only in the venom gland transcriptome (squares with green oblique lines and suffix “mRNA”).^this work,^ ^92^ Species in clades marked with red triangles express only crotoxin-negative (CTx-) venoms. Species within the clades identified with dark gray triangles do not express crotoxin-like molecules in their venoms, or these have not yet been investigated (NI) yet. *Gloydius intermedius* represents the Asian species, considered the closest extant descendant of the ancestor that carried the genes coding for the crotoxin-like heterodimeric PLA_2_ across the Bering Strait.

The crystal structure of crotoxin from *Crotalus durissus terrificus*, solved at 1.35 Å resolution,^93^ revealed that the posttranslational processing at two chymotryptic and one tryptic cleavage sites of the acidic subunit precursor to form three polypeptides held together by disulfide bridges is a prerequisite for the assembly of the native functional heterodimeric β-neurotoxin. It has been proposed that a single nucleotide change unlocked buried cleavage sites already present in the ancestral acidic PLA_2_ precursor, AncA2, enabling the proteolytic processing that exposed a latent hydrophobic interface for dimerization with ancestral basic PLA_2_ AncB2.^94^ The three proteolytic cleavage sites are conserved in Bnigro-PLA2-2 (= Bnubes-PLA2-2) (Figure 5A), and translated Bnigro-PLA2-2 assembles with the Bnigro-PLA2-1 processed protein into the crotoxin-like heterodimer Nigroviriditoxin,^40^ which represents 38% of the black-speckled palm pitviper, *Bothriechis nigroviridis*, venom proteome.^39^

**Figure 5:**
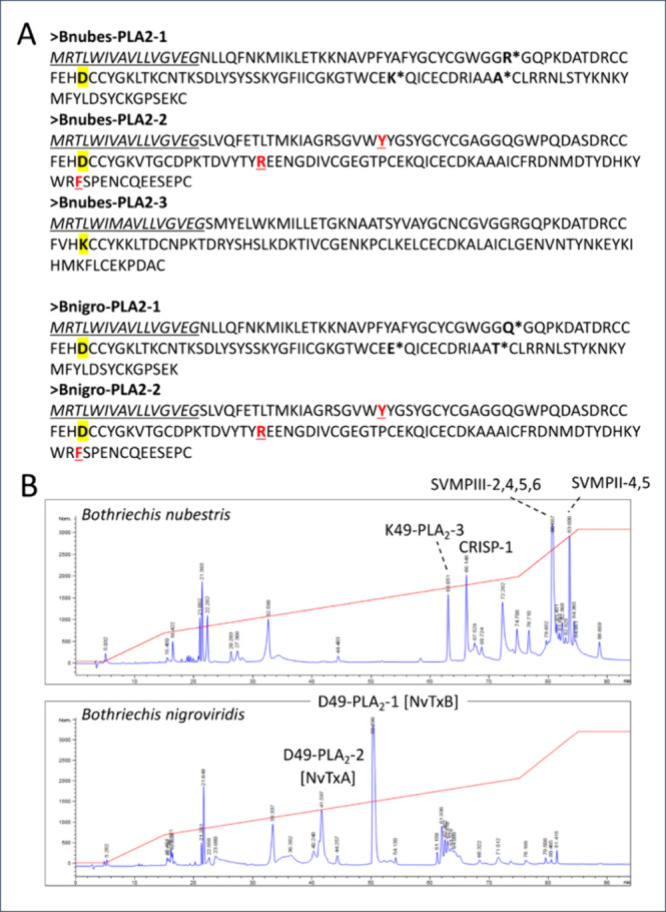
Convergent transcription and divergent translation in the venom glands of *B. nubestris*and *B. nigroviridis*. Panel A, amino acid sequences encoded in *B. nubestris* and *B. nigroviridis* venom gland transcriptomes. Signal sequences are in italics and underlined. D49 and K49 residues are shown in boldface and yellow background. Bnubes-PLA2-2 and Bnigro-PLA2 share identical primary structure. The three divergent residues (33R/Q, 83K/E, and 94A/T) in the amino acid sequences of Bnubes-PLA2-1 and Bnigro-PLA2-1 are highlighted in bold and labeled with asterisks. The conserved residues whose proteolytic cleavage is necessary for the assembly of the processed acidic (PLA2-2) subunit with the basic PLA2-1 subunit into heterodimeric nigroviriditoxin (Y20, R67, F110) are shown in boldface, in red, and underlined. Panel B, Comparative RP-HPLC profiles of *B. nubestris* and *B. nigroviridis* venoms highlighting remarkable divergent compositional features. NvTxA and NvTxB are acidic and basic subunits of nigroviriditoxin.

On the contrary, strikingly, identical Bnubes-PLA2-2 and Bnubes-PLA2-1 transcripts do not produce any translation product into the venom of the sister species, *B. nubestris* (Figure 5B). On the other hand, despite the fact that *B. nigroviridis* transcriptome contains full-length orthologues of *B. nubestris* PII- and PIII-SVMPs transcripts, these molecules were not found translated in the venom proteome of *B. nigroviridis* from the Central Highlands of Costa Rica (Figure 5B).^39^

High SVMP content and lack of heterodimeric crotoxin-like PLA_2_ of *B. nubestris* venom represent characteristics of type-I venoms, whereas the opposite phenotype observed in *B. nigroviridis* from the Central Highlands of Costa Rica, high level of heterodimeric crotoxin-like PLA_2_ and low content/absence of SVMPs, are typical features of type-II crotalid venoms.^87^ Pure venom phenotypic type-I/type-II dichotomies and variation within venom types^2,95^ have been documented between and within genera and species, and different underlying mechanisms have been proposed. Thus, the pattern of expression of type-I and type-II venom phenotypes in North American rattlesnake has been adscribed to recent lineage-specific gene loss within the PLA_2_ and SVMP multigene clusters, suggesting that venom phenotype was determined by the genomic presence or absence of SVMP and PLA_2_ gene family members.^96,97^ However, recent work on the genome of the Tiger Rattlesnake, *Crotalus tigris*,^98^ which possesses the simplest and most toxic venom of any rattlesnake species^99^ has shown that gene presence–absence explained some, but not all, of the variation within venom types.

The expression of type-II venoms throughout the life of Neotropical rattlesnakes (*Crotalus durissus* sp.) represents an adaptive paedomorphic trait driven by gain of neurotoxicity to rodents along the north–south axis of Crotalus radiation in South America.^13^ On the other hand, in the phylogenetically basal Central American rattlesnake *C. simus* clade the dichotomous phenotypes are regulated by an ontogenetic transition from type-II (juvenile) to type-I (adult) venom, and correlation between age-dependent changes in the concentration of venom gland miRNAs and the ontogenetic type II to type I venom shift occurring in Costa Rican and Mexican Central American rattlesnake *Crotalus simus*, and *C. tzabcan* (Yucatán neotropical rattlesnake) have been documented.^100,101^ Also, hybridization between a male Southern Pacific Rattlesnake (*Crotalus oreganus helleri*) with type-I venom and a female Mojave Rattlesnake (*Crotalus scutulatus scutulatus*) snake with type II venom, yielded offspring in which venom exhibited overlapping parental venom profile.^102^ Hybrid venom phenotypes that result from natural introgression between two species that express highly divergent venoms, *Crotalus o. concolor* (Midget Faded Rattlesnake, type-II venom) and *C. v. viridis* (Prairie Rattlesnake, type-I venom), provided further evidence that type-I and type-II venom characteristics can be expressed simultaneously in hybrid venoms.^103^

The crotoxin-type basic subunit is widespread among Crotalinae venoms, where its expression is independent of the acidic subunit and is present in 2.1–4.5-fold excess with respect to the A-subunit.^99^ Translation into the venom of a processed acidic subunit is the limiting factor for assembling a functional heterodimeric “nubestritoxin” molecule.^104^ The enzyme(s) responsible for the processing of the A-subunit remain elusive. Here, we have analyzed the primary structures encoded in the 9 and 13 SVSP transcripts of *B. nubestris* and *B. nigroviridis* transcriptomic data sets, respectively. Multiple sequence alignment identified the following orthologous protein groups (Bnubes:Bnigro): SVSP-6/SVSP-6; SVSP-1/SVSP-1; SVSP-2/SVSP-2; SVSP-8/SVSP-(8/12); SVSP-9/SVSP-(11/13); SVSP-4/SVSP-4; SVSP-5/SVSP-5; SVSP-7/SVSP-7; SVSP-3/SVSP-3. All the sequences contain the catalytic triad of serine proteinases (H57, D102, and S195, highlighted in boldface in ), suggesting that, if translated, the resulting proteins would be catalytically active. The substrate specificity of trypsin and chymotrypsin is primarily determined by the S1 site. Position 189, located at the base of the S1 pocket, is very highly conserved as an Asp in enzymes with trypsin-like specificity toward Arg- and Lys containing substrates, whereas it is found as a Ser or other small amino acid in chymotrypsin and elastase-class enzymes, which manifest specificity toward aromatic and small hydrophobic amino acids, respectively.^105,106^ With the exception of Bnigro-SVSP-6 (=Bnubes-SVSP-6), which has Gly189, all of the other *B. nubestris* and *B. nigroviridis* SVSPs have Asp in this S1 site position (). This evidence strongly suggests that *B. nubestris* and *B. nigroviridis* are nominally capable of synthesizing both trypsin-like and chymotrypsin-type serine proteinases.

BLAST analyses of the nine groups of orthologous SVSPs against the NCBI Serpentes data set showed extensive homology (>82% sequence identity; 89% sequence similarity) with a number of Asp189 SVSP from both type-I and type-II Crotalus and Gloydius venoms. Only the chymotrypsin-class SVSP-6 showed 88–94% sequence identity with Ser/Gly189 SVSPs *from Crotalus scutulatus*, *Crotalus tigris*, *Crotalus mitchelli*, *Crotalus durissus terrificus*, *Crotalus tzbacan*, and *Gloydius intermedius* (). It is thus tempting to hypothesize that SVSP-6 might be the enzyme responsible for the post-translational processing of the acidic subunit precursor at the chymotryptic cleavage sites.

Given that the transcriptomes of *B. nubestris* and *B. nigroviridis* share all the players necessary to express functional heterodimeric crotoxin-like PLA_2_, it seems thus reasonable to assume that the absolute absence of Bnubes-PLA2-1 and Bnubes-PLA2-2 in the venom proteome of the *B. nubestris* LIAP 0292 specimen investigated here can not be adscribed to gene loss. So, what limits *B. nubestris* to express nigroviriditoxin (=nubestritoxin) in its venom? Or what prevents *B. nigroviridis* from expressing SVMPs in its venom? The answer could lie in a differential post-transcriptional management of the conserved transcripts’ translation in the *B. nubestris* and *B. nigroviridis* venom glands. Potentially relevant to address this issue is the report of hypothetical dual-action miRNAs predicted to silence the translation of mRNAs for crotoxin A-subunit while simultaneously up-regulating SVMP-targeting mRNAs.^101^ To illustrate this point, Figure 6 displays the result of a preliminary (unpublished) proof-of-concept carried out to falsify the existence of dual-action miRNAs, which showed a small but consistent trend toward a decrease in the relative abundance of the crotoxin basic subunit (as a proxy for the decrease in heterodimer production) and a not conclusive increase in the relative abundance of PIII-SVMP. The evidence that miRNAs might modulate the differential expression of toxins suggests a mechanism to explain the distinct usage of highly similar transcriptomes to produce the dichotomic venom phenotype expressed by the closely related Costa Rican palm pitvipers.

**Figure 6:**
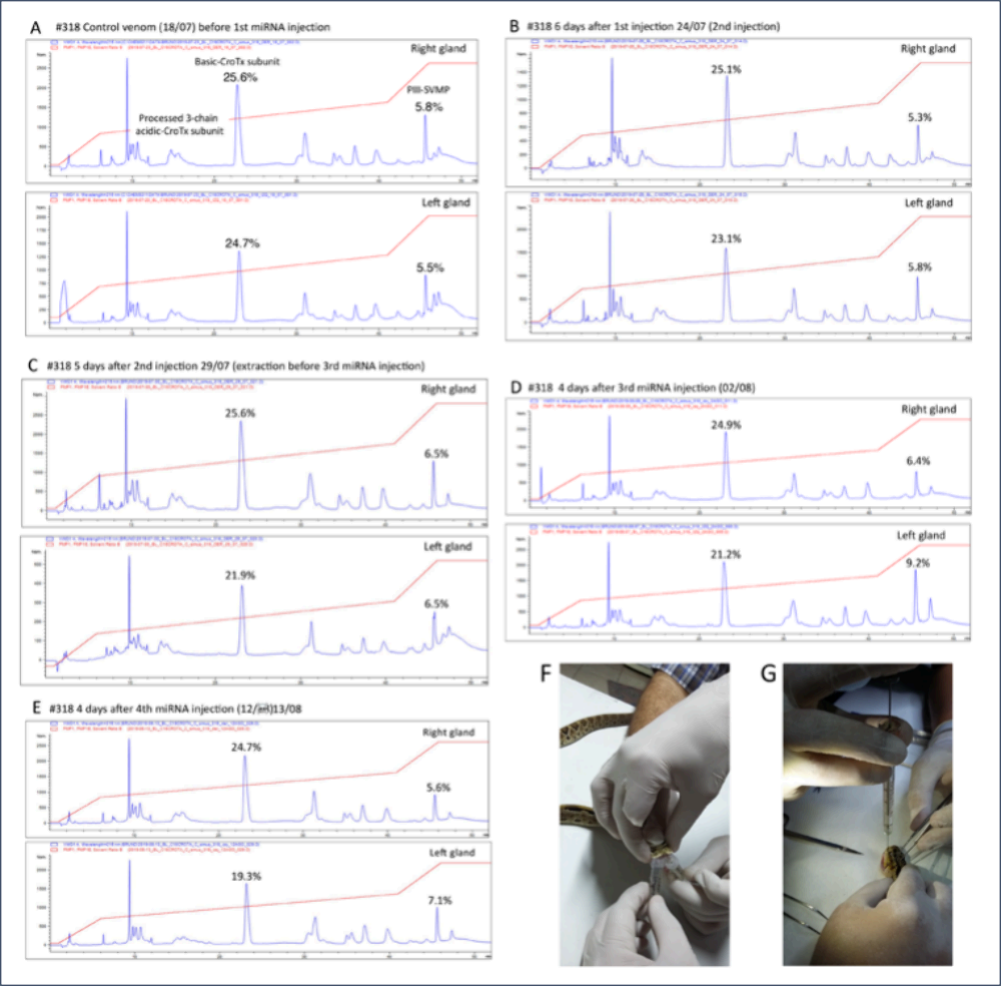
Effect of miRNAs on the translation of crotoxin-like heterodimeric PLA_2_s and PIII-SVMP in the venom of neonate Costa Rican *C. simus*. Twenty-five μg of a mixture of two putative dual-action miRNAs, CSA1063-1742 and CSA9692-235, predicted to target CTx A/P00623 and PIII Q2QA02 ( ^101^), in 0.9% NaCl were injected every third day into the left venom gland of a neonate *C. simus*, whereas the contralateral right gland received control buffer. Comparative reverse-phase HPLC chromatograms display profiles of venom obtained from the control (right) and the miRNA-treated (left) glands of neonate #318 *C. simus* before (Panel A) and after succesive injections of miRNA (Panels B–E). Panels F and G, Simultaneous venom extraction from the right and the left gland of neonate *C. simus* #318, and injection of miRNAs in the left gland, respectively.

## 4 Concluding Remarks and Future Perspectives

Miocene massive uplift along the mountain backbone from the Chiapan highlands in lower Mexico to the Talamancan highlands in Central Costa Rica initially separated the widespread lowland *B. schlegelii* from the montane taxa.^32,107^ Late Miocene–early Pliocene (13–5 Mya) orogenic activity and climatic fluctuations associated with the Nicaraguan Depression, the Motagua-Polochic fault, and the closure of the Panamanian Isthmus, changed topologies and imposed biogeographic breaks that promoted allopatric speciation.^34,36,108–110^ Reconstruction of ancestral *Bothriechis* distribution indicated that the ancestor of *B. schlegelii* and *B. supraciliaris* inhabited all of Middle America while the common ancestor of the remaining taxa was restricted to the region above the Isthmus of Panamá,^110^ until the final closure of this land bridge in the Pliocene, 2.8 to 2.6 Mya,^111^ when modern elevations in the Colombian Andes were reached,^112^ allowed *B. schlegelii* to radiate into South America.

The Nicaraguan Depression, extending for 800 km through El Salvador and western Nicaragua to the Caribbean Sea in northeastern Costa Rica, has been implicated in the separation of the Nuclear Middle American species (*B. aurifer*, *B. bicolor*, *B. guifarroi*, *B. marchi*, *B. rowleyi*, and *B. thalassinus*) from those species restricted to the southern Middle American Isthmus (*B. lateralis*, *B. nigroviridis*, *B. nubestris*, and *B. supraciliaris*).^108,110^ Lower Central American taxa (i.e., *B. guifarroi*, *B. lateralis*, *B. nigroviridis*) may have split from Nuclear Central American taxa (*B. marchi*, *B. thalassinus*, *B. aurifer*, *B. bicolor*, *B. rowleyi*) at the Nicaraguan Depression between 6 and 10 Mya.^36,108^ Similarly, the Motagua-Polochic strike-slip faulting system along the western North America–Caribbean plate boundary zone, which cuts across Guatemala^113^ and is thought to be responsible for the generation of a majority of the mountain-building across southwestern Mexico and Nuclear Central America,^114^ has been proposed as a break dividing the Mayan block clade (*B. aurifer*, *B. bicolor*, *B. rowleyi*) from the Chortis block clade (*B. guifarroi*, *B. marchi*, and *B. thalassinus*) 10–8.5 Mya, concordant with the timing of the west to east progression of the Chortis block along the Mayan block to its current position.^39^ The divergence of the *B. nigroviridis*–*B. nubestris* lineage from the other *Bothriechis* has been dated at 13–7.5 Mya as the result of a shift to higher elevation habitats (*B. nigroviridis* from 500 to 2300 m to 1200–3000 m; *B. nubestris* from 2100 m (Cerro de la Muerte) to close to 3000 m (San Gerardo de Dota) on the Costa Rican Talamanca Mountain Range.^26,39^ However, it is still elusive how these species diverged from a common ancestor in the mountains of Costa Rica. Differential dispersal in different environments mediated by the climatic variation experienced in the region may have promoted the allopatric differentiation of these lineages.

Figure 7 displays a cartoon of the dated phylogenetic tree of the genus *Bothriechis* including the venom compositions known to date.

**Figure 7:**
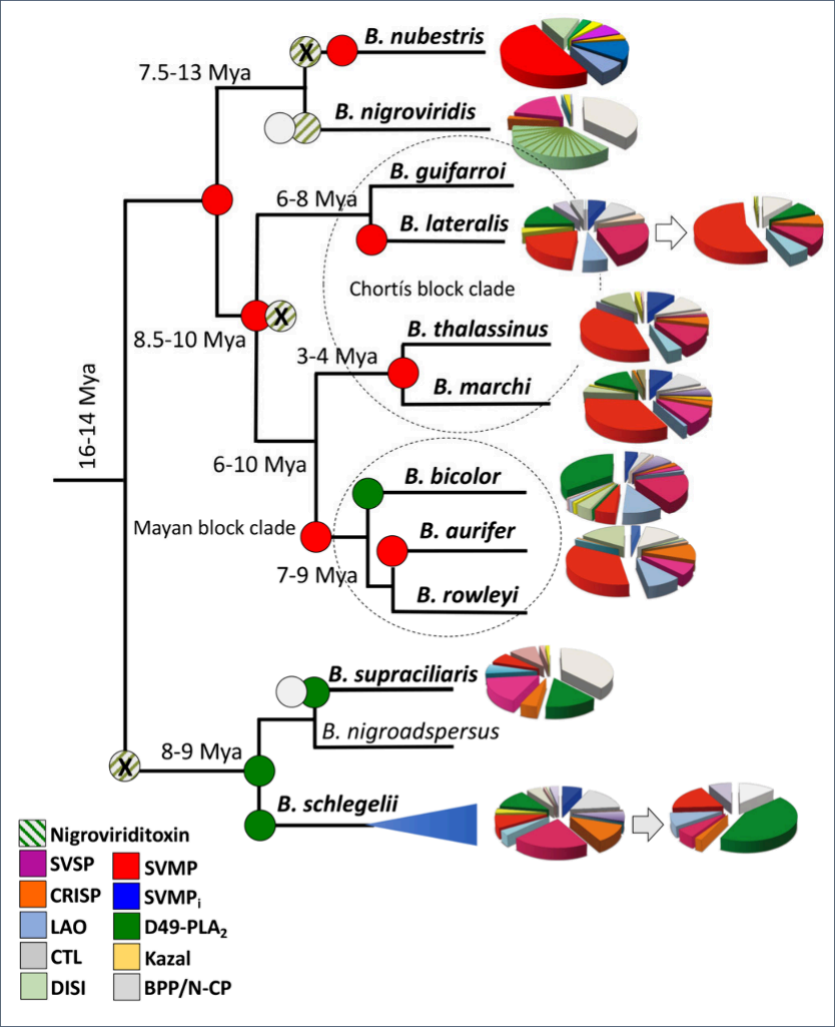
Venom variability across the genus *Bothriechis*. Scheme of phylogenetic relationships within the Bothriechis clade (based on data published by Alencar et al., 2016;^30^ Mason et al., 2019;^36^ Reyes-Velasco, 2024;^28^ Arteaga et al., 2024 ^27^) highlighting the remarkable venom divergence landscape displayed in pie chart format showing the relative abundance of toxin classes in the color coding shown at the below left corner of the figure. The 11 recognized species are highlighted in boldface. Estimated mean divergence times for selected nodes are indicated millions of years ago (mya). Colored circles represent nodes along the evolutionary distribution routes of major toxin classes across palm-pitviper cladogenesis: SVMP (red), PLA_2_ (green), BPP (gray), crotoxin-like heterodimeric PLA_2_ (diagonal pale green lines). An “X” at the crotoxin-like heterodimeric PLA_2_ node denotes loss of expression of this toxin. Arrows indicate ontogenetic venom compositional shifts reported for *B. lateralis* and *B. schlegelii* (Pla et al., 2017 ^18^). The blue horizontal triangle includes nominal *B. schlegelii* and its proposed geographic synonyms or subspecies. Acronym keys are the same as in the legend of Figure 2.

Type-I/type-II venom dichotomy^20,95,115,116^ within the Mojave-Sonoran clade of *C. scutulatus* has been strongly correlated with environmental conditions, with type-I venom areas characterized by larger diurnal thermal fluctuations, milder winters and less seasonal variation in precipitation.^24^ Climatic variables may be linked to prey distributions and snake physiology, which, in turn, impose selection pressures on snake venoms. In this sense, microRNAs are small (21–23-nucleotide long) noncoding RNAs that target complementary sites in the 3′UTR of their target mRNAs, thereby recruiting a complex of proteins that ultimately guide endoribonuclease Argonaute (Ago) to direct target degradation or alternatively down-regulate^117–119^ or activate^120–122^ translation. Alterations of the distribution of miRNAs, modulating the translational activity of venom gland toxin-encoding mRNAs in response to an external cue, could represent an underlying mechanism for the venom type-I/type-II phenotypic dichotomy. From an ecological-evolutionary perspective, a post-transcriptional mechanism that modulates rapid variation of the venom phenotype can potentially confer adaptive advantages in response to environmental changes. We hope that our work will spark interest in future studies that address the extent of this mechanism among species in which the occurrence of crotoxin-like heterodimeric PLA_2_ molecules have been shown in their venom or in the venom gland transcriptome (Figure 4).

## Supplementary Material







## Data Availability

Mass spectrometry data have been deposited with the MassIVE repository under accession number MSV000096598 (ftp://massive.ucsd.edu/MSV000096598/) and in ProteomeXchange with accession number PXD058628.

## Footnotes

1 Venom was analyzed from a single specimen and therefore the reported venom phenotype might be unique to the single individual sampled rather than representing the species as a whole. Intra- and interpopulation-level sampling is needed to define the degree of venom variation and the ecological correlations underlying such variability.

## Notes

**Supplementary File S1**: Multiple sequence alignment of the snake venom serine proteinases (SVSP) translated from the venom gland transcriptomes of *Bothriechis nigroviridis* and *Bothriechis nubestris* (PDF)
**Supplementary File S2**: BLAST analysis of the serine proteinases (SVSP) transcripts gathered in the venom gland transcriptomes of *Bothriechis nubestris* (Bnubes) and *Bothriechis nigroviridis* (Bnigro). S1 Asp189 residue s highly conserved in enzymes with trypsin-like specificity toward Arg- and Lys containing substrates. This position is a Ser or other small amino acid (mostly Gly) in chymotrypsin and elastase-class enzymes which manifest specificity toward aromatic and small hydrophobic amino acids, respectively. With the exception of Bnigro-SVSP-6 (=Bnubes-SVSP-6), which has Gly189, all the other *B. nubestris* and *B. nigroviridis* SVSPs have Asp in this S1 site position (PDF)
Supplementary Tables S1–S3: **Supplementary Table S1**: Peptides and proteins matched in *B. nubestris* venom in the *B. nubestris* and *B. nigroviridis* databases **Supplementary Table S2**: Peptides gathered by bottom-up peptidomics and venomics of reduced and carbamidomethylated *B. nubestris* SDS-PAGE bands of venom fractions, and their match in the transcriptomic databases of *B. nubestris* and *B. nigroviridis*. **Supplementary Table S3**: Relative quantification of *B. nubestris* venom peptidome and proteome (XLSX)
