# Supplementary material for: Snake Venomics of the Arboreal Talamancan Palm-Pitviper, Bothriechis nubestris, Provides Clues on the Origin of a Phenotypic Dichotomy between Type‑I and Type-II Venoms
Source: J Proteome Res. 2025 Apr 25;24(6):2801–15. doi: 10.1021/acs.jproteome.4c01041 (PMC12150329; doi:10.1021/acs.jproteome.4c01041)
Supplement: Supplementary file 1 [file pr4c01041_si_001.pdf]

Supplementary File S1: CLUSTAL O(1.2.4) multiple sequence alignment of the snake venom serine proteinases (SVSP) translated from the venom gland transcriptomes of *Bothriechis nigroviridis* (Bnigro) and *Bothriechis nubestris* (Bnubes) (Mason et al. 2020). Catalytic triad amino acids (H57, D102, S195) are highlighted in boldface. Residues Asp189, conserved in enzymes with trypsin-like specificity, and Ser189 and W173, found in chymotrypsin- and elastase-class enzymes, which manifest specificity toward aromatic and small hydrophobic amino acids, are in red and in green, respectively.

Bnigro-SVSP-6   MVLIRVLANLLILQLSYAQKSSELVIGGDECNRNEHRSLALVY--ITSGFLCAGTLINQE 58
Bnubes-SVSP-6   MVLIRVLANLLILQLSYAQKSSELVIGGDECNINEHRSLALVY--ITSGFLCAGTLINQE 58
Bnigro-SVSP-1   MVLIRVLANLLILQLSYAQKSSELVVGGGECNINEHRSLVAFF--NSTGFFCSGTLINEE 58
Bnubes-SVSP-1   MVLIRVLANLLILQLSYAQKSSELVVGGDECNINEHRSLVAIF--NSTGFFCSGTLINEE 58
Bnigro-SVSP-9   MVLIRVLANLLILQLSYAQKSSELVVGGHPCNINEHRSLVVLF--NSSGFFCAGTLINQE 58
Bnigro-SVSP-2   MVLIRVLANLLILQLSYAQKSSKLVVGGDECNINEHRSLVVLF--NSSSFLCGGTLINKE 58
Bnubes-SVSP-2   MVLIRVLANLLILQLSYAQKSSKLVVGGDECNINEHRSLVVLF--NSSSFLCGGTLINKE 58
Bnigro-SVSP-8   MVLIRVLANLLILQFSYAQKSSELIIGGDECNINEHRFLVALYHSMSKTFLCGGTLINEE 60
Bnubes-SVSP-8   MVLIRVLANLLILQFSYAQKSSELIIGGDECNINEHRFLVALYHSMSKTFLCGGTLINEE 60
Bnigro-SVSP-12   MVLIRVLANLLILQFSYAQKSSELVIGGDECNINEHRFLVALYHSRSKTFLCGGTLINEE 60
Bnigro-SVSP-11   MVLIRVLANLLILQLSYAQKSSELIIGGGECNINEHRFLVAM--YGPNGFLCGGTLINKE 58
Bnigro-SVSP-13   MVLIRVLVNLLILQLSYAQKSSELVIGGGECNINEHRFLVALHDYWSGSFLCGGTLINKE 60
Bnubes-SVSP-9   MVLIRVLANLLILQFSYAQKSSELVIGGDECNINEHRFLVALYDYWSGSFLCGGTLINKE 60
Bnigro-SVSP-4   MVLIRVLANLLILQISYAQKSSELIIGGDECNINEHRSLVLVNF---DGFQCAGTLINQE 57
Bnubes-SVSP-4   MVLIRVLANLLILQISYAQKSSELIIGGDECNINEHRSLVLVNF---DGFQCAGTLINQE 57
Bnigro-SVSP-5   MVLIRVLANLLILQFSYAQKSSELVVGGDECNINEHRSLVLVYS---DGIQCGGTLINQE 57
Bnubes-SVSP-5   MVLIRVLANLLILQFSYAQKSSELVVGGDECNINEHRSLVLVYS---DGIQCGGTLINQE 57
Bnigro-SVSP-7   MVLIRVLANLLILQLSYAQKSSELIIGGDECNINEHRFLALVYS---DRFQCGGTLINEE 57
Bnubes-SVSP-7   MVLIRVLANLLILQLSYAQKSSELIIGGDECNINEHRFLALVYS---DRFQCGGTLINEE 57
Bnigro-SVSP-10   MVLIRVLANLLILQLSYAQKSSELIIGGGECNINEHRFLVALYTFT--RFLCGGTLINQE 58
Bnigro-SVSP-3   MVLIRVLANLLILQFSYAQKSSELIIGGDECNINEHRFLVALYTFRSRRFHCSGTLINQE 60
Bnubes-SVSP-3   MVLIRVLANLLMLQFSYAQKSSELIIGGDECNINEHRFLVALYTFRSRRFHCSGTLINQE 60
                    *******.***:**:*******:*::** ** **** *. .   : *.*****:*

Bnigro-SVSP-6   WVLTAA**H**CNRGNIHIFLGVHSLKVLNKDKPTRIAKEKFICPNRKKDDEKDK**D**IMLIRLDS 118
Bnubes-SVSP-6   WVLTAA**H**CDRGNIHIFLGVHSLKVLNKDKPTRIAKEKFICPNRKKDDEKDK**D**IMLIRLDS 118
Bnigro-SVSP-1   WVLSAA**H**CDSTNFQIKLGVHSKKALNEDEQTRNPKEKFICPNKKNDEILDK**D**IMLIKLDS 118
Bnubes-SVSP-1   WVLTAA**H**CDSTNFQMKFGVHSKKALNEDEQTRNPKEKFICPNKKNDDVLDK**D**IMLIKLDS 118
Bnigro-SVSP-9   WVLTAA**H**CDIKNFQIQLGVHNKKVRNEDEQTRDPKEKFFCLSSKTNNEWDK**D**IMLIRLDS 118
Bnigro-SVSP-2   WVLTAA**H**CDSKNFQMMFGVHSKKVLNEDEQTRDPKEKFICPNRKKDDERDK**D**IMLIRLDS 118
Bnubes-SVSP-2   WVLTAA**H**CDSKNFQMMFGVHSKKVLNEDEQTRDPKEKFICSNRKKDDERDK**D**IMLIRLDS 118
Bnigro-SVSP-8   WVLTAS**H**CNRLFMYIKLGMHNKNVKFDDEQRRYPKEKYFFRCHNNFTRWDK**D**IMLIRLNS 120
Bnubes-SVSP-8   WVLTAS**H**CNRLFMYIKLGMHNKNVKFDDEQRRYPKEKYFFRCHNNFTRWDK**D**IMLIRLNS 120
Bnigro-SVSP-12   WVLTAA**H**CNRVFMYIKLGMHNKNVKFDDEQRRYPKEKYFFRCHNNFTKWDK**D**IMLIRLNS 120
Bnigro-SVSP-11   WVLTAA**H**CDRRNIYIYLGMHNQSVQFDDEQRRYPKEKYLFPCSKNFTKWDK**D**IMLIRLNR 118
Bnigro-SVSP-13   WVLTAA**H**CNRSNILIYLGVHNQSVQFDDEQRRYPKEKYLFRCSKTYTKWDK**D**IMLIKLNK 120
Bnubes-SVSP-9   WMLTAA**H**CNRSNILIYLGVHNQSVQFDDEQRRYPKEKYLFRCSKIFTKWDK**D**IMLIKLNK 120
Bnigro-SVSP-4   WVLSAA**H**CDGKKMKLQFGLHSKNVPNKDKQTRVAKETFFCLSSKNYTKWDK**D**IMLIRLNR 117
Bnubes-SVSP-4   WVLSAA**H**CDGKKMKLQFGLHSKNVPNKDKQTRVAKEKFFCLSSKNYTKWDK**D**IMLIRLNR 117
Bnigro-SVSP-5   WMLTAA**H**CDGKKMKLQFGLHSKNVPNKDKQTRVPKEKFFCLSSKNYTKWDK**D**IMLIRLNH 117
Bnubes-SVSP-5   WMLTAA**H**CDGKKMKLQFGLHSKNVPNKDKQTRVPKEKFFCLSSKNYTKWDK**D**IMLIRLNH 117
Bnigro-SVSP-7   WVLTAA**H**CDMRYMHIYLGVHNLSVKYDDEQRRFPKKKYFCLSSRNYTKWDK**D**IMLIRLNR 117
Bnubes-SVSP-7   WVLTAA**H**CDMRYMHIYLGVHNLSVKYDDEQRRFPKKKYFCLSSRNYTKWDK**D**IMLIRLNR 117
Bnigro-SVSP-10   WVLTAA**H**CDRRNIRIKFGMHSKKVLNEDEQTRVPKEKFFCLSSKNYTKWDK**D**IMLIRLNS 118
Bnigro-SVSP-3   WVLTAA**H**CDRKNIRIKLGMHSTNVTNEDKQTRVPKEKFFCLSSKNYTKWDK**D**IMLIRLKR 120
Bnubes-SVSP-3   WVLTAA**H**CDRKNIRIKLGMHSTNVTNEDKQTRVPKEKFFCLSSKTYTKWDK**D**IMLIRLKR 120
                    ::*:**:   : : :*:*. .. .*: * *:.::   .   *******:*.

Bnigro-SVSP-6   PVSNSEHIAPLSLPSNPPSVGSVCRVMGWGAITSPNVTLPGVPHCANINILDYEVCRAAY 178
Bnubes-SVSP-6   PVNNSTHIAPLSLPSNPPSVGSVCRVMGWGAITSPNVTLPGVPHCANINILDYEVCRAAY 178
Bnigro-SVSP-1   RVSNSEHIAPLSLPSSPPIVGSVCHIMGWGSITPIKVTYPDVPYCAYINLFDDAVCQPGY 178
Bnubes-SVSP-1   RVSNSEHIAPLSLPSSPPSVGSVCHIMGWGSITPIKVTYPDVPYCANINLLDDAVCQPGY 178
Bnigro-SVSP-9   SVSNSPHIAPLSLPSSPPSVGSLCRIMGWGTISSTKETYPNVPRCANINILDYAVCRAAY 178
Bnigro-SVSP-2   PVSNSEHIAPLSLPSNPPSVGSVCRIMGWGTISPTKETYPDVPHCANINILDHEVCQAAY 178
Bnubes-SVSP-2   PVSNSEHIAPLSLPSNPPSVGSVCRIMGWGTISPTKETYPDVPHCANINILDHEVCQAAY 178
Bnigro-SVSP-8   PVNNSTHIVPLSLPSSPPSVGSVCRVMGWGTTTSPQETLPDVPRCANINLVNYTMCRGVF 180
Bnubes-SVSP-8   PVNNSTHIVPLSLPSSPPSVGSVCRVMGWGTTTSPQETLPDVPRCANINLVNYTMCRGVF 180
Bnigro-SVSP-12   PVNNSTHIVPLSLPSSPPSVGSVCRVMGWGTTTSPQETLSDVPRCANINLVNYTMCRGVF 180
Bnigro-SVSP-11   PVRKSAHIAPLSLPSNPPSVGSVCRVMGWGTITSPNETLPDVPRCANINLFNYTECRGVF 178
Bnigro-SVSP-13   PVRNSAHIAPLGLPSSPPIVGSVCRVMGWGSITSPKETLPDVPRCANINLLNYTECCGIY 180
Bnubes-SVSP-9   PVRNREHIAPLSLPSSPPIVGSVCRVMGWGTITSPKETLPDVPRCANINLLNYTECRGIY 180
Bnigro-SVSP-4   PVNNSTHIAPLSLPSNPPSVGSVCRIMGWGTISSTKVNLPDVPHCANINIIDYEVCRTAY 177

```
Bnubes-SVSP-4       PVNNSTHIAPLSLPSNPPSVGSVCRIMGWGTISSTKVNLPDVPHCANINIIDYEVCRTAY 177
Bnigro-SVSP-5       PVNNSTHIAPLSLPSNPPSQDTVCNIMGWGTISASKEIYPDVPHCANINILNNAVCRPIY 177
Bnubes-SVSP-5       PVNNSTHIAPLSLPSNPPSQDTVCNIMGWGTISASKEIYPDVPHCANINILNNAVCRPIY 177
Bnigro-SVSP-7       PVRNSAHITPLSLPSNPPSVGSVCRVMGWGTITSPNVTYPDVPYCANINLLDYEVCRAAY 177
Bnubes-SVSP-7       PVRNSAHITPLSLPSNPPSVGSVCRVMGWGTITSPNVTLPDVPHCANINILDYEVCRAAY 177
Bnigro-SVSP-10      PVNNSTHIAPLSLPSNPPIVGSVCRIMGWGTITSPNVTLPDVPHCANINLFNYTVCHGAH 178
Bnigro-SVSP-3       PVNKSIHIAPVSLPSNPPSLGSVCRIMGWGTISATKETYPKVPHCANINILDYEVCRGAF 180
Bnubes-SVSP-3       PVNKSIHIAPVSLPSNPPSLGSVCRIMGWGTISATKETYPEVPHCANINILDYEVCRGAF 180
                     * :  **.*:.***.**  .::*.:****: :  :     ** ** **:.:   *   .


Bnigro-SVSP-6       P--LWPATRRTLCAGILEGGKGSCDGDSGGPLICNGEIQGIISWGGDICAQPREPGHYTK 236
Bnubes-SVSP-6       P--QWPATRRTLCAGILEGGKGSCDGDSGGPLICNGEIQGIVSWGGDICAQPREPGHYTK 236
Bnigro-SVSP-1       P--ELLPEYRTLCAGIVQGGKDTCGGDSGGPLICNGQFQGIVSYGAHPCGQDIKPGVYTK 236
Bnubes-SVSP-1       P--EVLPEYRTLCAGIVQGGKDTCGGDSGGPLICNGQFQGIVSYGAHPCGQDLKPGIYTK 236
Bnigro-SVSP-9       P--WWPATTTKLCAGILEGGKDSCQGDSGGPLICNGEFQGIVSWGPHPCGRRLKPGFYTK 236
Bnigro-SVSP-2       P--WQPVTSTTLCAGILEGGKDTCHADSGGPLICNGQFQGIVSWGVHPCAQSHAPGVYTK 236
Bnubes-SVSP-2       P--WQPVTSTTLCAGILEGGKDTCHGDSGGPLICNGQFQGIVSWGGHPCGQLLEPGLYTK 236
Bnigro-SVSP-8       P--KLPERSRTLCAGVLEGGIDTCNRDSGGPLICNGQFQGIVFWGRNPCAQPREPALYTK 238
Bnubes-SVSP-8       P--TLPERSRTLCAGVLEGGIDTCNRDSGGPLICNGQFQGIVFWGRDPCGQPREPALYTK 238
Bnigro-SVSP-12      P--TLPERSRTLCAGVLEGGIDTCHRDSGGPLICNGQFQGILSWGWYPCAQPRKPALYSK 238
Bnigro-SVSP-11      P--RLPARSRTLCAGVLEGGIDTCNRDSGGPLICNGQFQGIVSWGPDPCAQPRKPALYSK 236
Bnigro-SVSP-13      P--ELPARSRMLCAGVLEGGIDTCNHDSGGPLICNGQFQGILSWGWYPCAQPRKPALYSK 238
Bnubes-SVSP-9       P--ELPARSRMLCAGVLEGGIDTCNHDSGGPLICNGQFQGILSWGWYPCAQPRKPALYSK 238
Bnigro-SVSP-4       PQYGLPATSRTLCAGILEGGKDTCVGDSGGPLICNGQFQGIVSWGSDVCGYIREPALYTK 237
Bnubes-SVSP-4       PQYGLPATSRTLCAGILEGGKDTCVGDSGGPLICNGQFQGIVSWGSDVCGYIREPALYTK 237
Bnigro-SVSP-5       S--GLLEKSKTLCAGILEGGKDTCGGDSGGPLICNGQIQGILSVGGDPCALPHVPALYTK 235
Bnubes-SVSP-5       S--GLLEKSKTLCAGILEGGKDTCGGDSGGPLICNGQIQGILSVGGDPCALPHVPALYTK 235
Bnigro-SVSP-7       P--ELPAKRKTLCAGILEGSKDSCDGDSGGPLICNGQFQGIVSWGADTCAQPREPGLYTK 235
Bnubes-SVSP-7       P--ELPAKRKTLCAGILEGSKDSCDGDSGGPLICNGQFQGIVSWGADTCAQPREPGLYTK 235
Bnigro-SVSP-10      A--GLPATSRTLCAGIVQGGIDSCKGDSGGPLICNGQFQGIVSWGGDPCAQPHEPGLYTK 236
Bnigro-SVSP-3       P--WLPATSRTLCAGILEGGKDSCKGDSGGPLICNGEIQGIVSWGGDPCALPRVPGLYTK 238
Bnubes-SVSP-3       P--WLPATSRTLCAGILEGGKDSCKGDSGGPLICNGQFHGIVSWGGDPCALPRVPGLYTK 238
                               ****:::*. .:*  **********:::**:  *   *.    *. *:*

Bnigro-SVSP-6       VFDYIDWIQSIIAGNTDATCPP  258
Bnubes-SVSP-6       VFDYTEWIQSIIAGNTDATCPP  258
Bnigro-SVSP-1       VFDYNDWIQSIIAGNTAATCPP  258
Bnubes-SVSP-1       VFDYNDWIQSIIAGNTAATCPP  258
Bnigro-SVSP-9       VFDYIDWIQSIIAGNTRVTCPQ  258
Bnigro-SVSP-2       VFDYTDWIQSIIAGNTTATCPL  258
Bnubes-SVSP-2       VFDYTEWIQSIIAGNTDATCPL  258
Bnigro-SVSP-8       VFDHLDWIQSIIAGSKTVTCPP  260
Bnubes-SVSP-8       VFDHLDWIQSIIAGSKTVTCPP  260
Bnigro-SVSP-12      VFDHLDWIQSIIAGSKTVTCPP  260
Bnigro-SVSP-11      VFDHLDWIQSIIAGNKTVTCPP  258
Bnigro-SVSP-13      VFDHLDWIQSIIAGNKTVTCPP  260
Bnubes-SVSP-9       VFDHLDWIQSIIAGSKTVTCPP  260
Bnigro-SVSP-4       VLDYTDWIQSIIAGNTNATCPP  259
Bnubes-SVSP-4       VLDYTDWIQSIIAGNTNATCPP  259
Bnigro-SVSP-5       VFDYTEWIQSIIAGNTDVACLP  257
Bnubes-SVSP-5       VFDYTEWIQSIIAGNTDAACLP  257
Bnigro-SVSP-7       VFDYIDWIQSIISGNNNATCPP  257
Bnubes-SVSP-7       VFDYIDWIQSIISGNNNATCPP  257
Bnigro-SVSP-10      VFDYTEWIQSIIAGNTTATCPP  258
Bnigro-SVSP-3       VFDYTEWIQSIIAGNADATCPP  260
Bnubes-SVSP-3       VFDYTEWIQSIIAGNADATCPP  260
                    *:*: :******:*.  .:*
```
