# Supplementary material for: Snake Venomics of the Arboreal Talamancan Palm-Pitviper, Bothriechis nubestris, Provides Clues on the Origin of a Phenotypic Dichotomy between Type‑I and Type-II Venoms
Source: J Proteome Res. 2025 Apr 25;24(6):2801–15. doi: 10.1021/acs.jproteome.4c01041 (PMC12150329; doi:10.1021/acs.jproteome.4c01041)
Supplement: Supplementary file 2 [file pr4c01041_si_002.pdf]

Supplementary File S2.

BLAST analysis of the serine proteinases (SVSP) transcripts gathered in the venom gland transcriptomes of *Bothriechis nubestris* (Bnubes) and *Bothriechis nigroviridis* (Bnigro).

>Bnubes SVSP-6/Bnigro SVSP-6

VIGGDECNINEHRSLALVYITSGFLCAGTLINQEWVLTAAHCDRGNIHIFLGVHSLKVLNKDKP
TRIAKEKFICPNRKKDDEKDKDIMLIRLDSPVNNSTHIAPLSLPSNPPSVGSVCRVMGWGAITS
PNVTLPGVPHCANINILDYEVCRAAYPQWPATRRTLCAGILEGGK**G**SCDGDSGGPLICNGEIQG
IVSWGGDICAQPREPGHYTKVFDYTEWIQSIIAGNTDATCPP

serine endopeptidase [Crotalus scutulatus] **S189**
Sequence ID: AUS82553.1 210/234(90%) 220/234(94%)
snake venom serine protease isoform X4 [Crotalus tigris] **S189**
Sequence ID: XP_039181652.1 211/234(90%) 218/234(93%)
snake venom serine protease isoform X3 [Crotalus tigris] **S189**
Sequence ID: XP_039181651.1 211/234(90%) 218/234(93%)
serine endopeptidase [Crotalus mitchellii] **S189**
Sequence ID: AUS82523.1 194/216(90%) 200/216(92%)
snake venom serine protease isoform X2 [Crotalus tigris] **S189**
Sequence ID: XP_039181650.1 210/234(90%) 220/234(94%)
snake venom serine protease isoform X1 [Crotalus tigris] **S189**
Sequence ID: XP_039181649.1 211/234(90%) 218/234(93%)
snake venom serine protease isoform X2 [Crotalus tigris] **S189**
Sequence ID: XP_039181647.1 210/234(90%) 220/234(94%)
serine endopeptidase [Crotalus tigris] **S189**
Sequence ID: AUS82559.1 207/234(88%) 217/234(92%)
snake venom serine protease isoform X1 [Crotalus tigris] **S189**
Sequence ID: XP_039181646.1 207/234(88%) 218/234(93%)
serine endopeptidase [Crotalus scutulatus] **G189**
Sequence ID: AUS82552.1 209/237(88%) 218/237(91%)
serine endopeptidase [Crotalus scutulatus] **G189**
Sequence ID: AUS82554.1 206/234(88%) 217/234(92%)
serine protease 4 [Crotalus tzabcan] **G189**
Sequence ID: ANN23912.1 193/220(88%) 203/220(92%)
serine protease 3 [Gloydius intermedius] **S189**
Sequence ID: ASX97877.1 210/234(90%) 217/234(92%)
RecName: Full=Snake venom serine protease;
Precursor [Crotalus durissus durissus] **G189**
Sequence ID: Q2QA04.1 209/238(88%) 219/238(92%)
serine endopeptidase [Crotalus molossus] **G189**
Sequence ID: AUS82535.1 196/220(89%) 203/220(92%)
serine endopeptidase [Crotalus lepidus] **S189**
Sequence ID: AUS82506.1 190/216(88%) 198/216(91%)

>Bnubes SVSP-1/Bnigro SVSP-1

VVGGGECNINEHRSLVAFFNSTGFFCSGTLINEEWVLSAAHCDSTNFQIKLGVHSKKALNEDEQ
TRNPKEKFICPNKKNDEILDKDIMLIKLDSRVSNSEHIAPLSLPSSPPIVGSVCHIMGWGSITP
IKVTYPDVPYCAYINLFDDAVCQPGYPELLPEYRTLCAGIVQGGKDTCGGDSGGPLICNGQFQG
IVSYGAHPCGQDIKPGVYTKVFDYNDWIQSIIAGNTAATCPP

venom plasminogen activator precursor 3 [Gloydius intermedius]
Sequence ID: ASX97882.1 217/234(93%) 225/234(96%) **D189**

snake venom serine protease catroxase-2 isoform X3 [Crotalus tigris]
Sequence ID: XP_039181639.1 202/234(86%) 212/234(90%) **D189**
snake venom serine protease catroxase-2 isoform X1 [Crotalus tigris]
Sequence ID: XP_039181637.1 202/234(86%) 212/234(90%) **D189**
serine endopeptidase [Crotalus mitchellii]
Sequence ID: AUS82525.1 201/234(86%) 211/234(90%) **D189**
serine endopeptidase [Crotalus scutulatus]
Sequence ID: AUS82541.1 199/234(85%) 212/234(90%) **D189**

>Bnubes SVSP-2/Bnigro SVSP-2
VVGGDECNINEHRSLVVLFNSSSFLCGGTLINKEWVLTAAHCDSKNFQMMFGVHSKKVLNEDEQ
TRDPKEKFICSNRKKDDERDKDIMLIRLDSPVSNSEHIAPLSLPSNPPSVGSVCRIMGWGTISP
TKETYPDVPHCANINILDHEVCQAAYPWQPVTSTTLCAGILEGGKDTCHGDSGGPLICNGQFQG
IVSWGGHPCGQLLEPGLYTKVFDYTEWIQSIIAGNTDATCPL

venom plasminogen activator precursor 2 [Gloydius intermedius]
Sequence ID: ASX97881.1 206/233(88%) 214/233(91%) **D189**
serine endopeptidase [Crotalus mitchellii]
Sequence ID: AUS82518.1 204/233(88%) 213/233(91%) **N189**
serine endopeptidase [Crotalus lepidus]
Sequence ID: AUS82511.1 211/233(91%) 219/233(93%) **N189**
serine endopeptidase [Crotalus atrox]
Sequence ID: AUS82489.1 211/233(91%) 219/233(93%) **D189**

>Bnubes SVSP-8/Bnigro SVSP-8
IIGGDECNINEHRFLVALYHSMSKTFLCGGTLINEEWVLTASHCNRLFMYIKLGMHNKNVKFDD
EQRRYPKEKYFFRCHNNFTRWDKDIMLIRLNSPVNNSTHIVPLSLPSSPPSVGSVCRVMGWGTT
TSPQETLPDVPRCANINLVNYTMCRGVFPTLPERSRTLCAGVLEGGIDTCNRDSGGPLICNGQF
QGIVFWGRDPCGQPREPALYTKVFDHLDWIQSIIAGSKTVTCPP

serine proteinase 5 [Crotalus adamanteus] **D189**
Sequence ID: AEJ31999.1 200/238(84%) 211/238(88%)
salmobin [Gloydius halys] **D189**
Sequence ID: AAC61838.1 187/236(79%) 208/236(88%)
snake venom serine proteinase 9 [Crotalus tigris] **D189**
Sequence ID: XP_039181644.1 199/238(84%) 213/238(89%)
serine endopeptidase [Crotalus scutulatus] **D189**
Sequence ID: AUS82547.1 198/238(83%) 214/238(89%)
thrombin-like enzyme crotalase [Crotalus tigris] **D189**
Sequence ID: XP_039181661.1 183/238(77%) 202/238(84%)
thrombin-like enzyme crotalase [Crotalus tigris] **D189**
Sequence ID: XP_039181661.1 183/238(77%) 202/238(84%)

> Bnigro SVSP-9/Bnigro SVSP-(11/13)
VIGGDECNINEHRFLVALYDYWSGSFLCGGTLINKE
WMLTAAHCNRSNILIYLGVHNQSVQFDDEQRRYPKEKYLFRCSKIFTKWDKDIMLIKLNK
PVRNREHIAPLSLPSSPPIVGSVCRVMGWGTITSPKETLPDVPRCANINLLNYTECRGIY
PELPARSRMLCAGVLEGGIDTCNHDSGGPLICNGQFQGILSWGWYPCAQPRKPALYSKVF
DHLDWIQSIIAGSKTVTCPP

venom plasminogen activator precursor 2 [Gloydius intermedius]
Sequence ID: ASX97881.1 206/233(88%) 214/233(91%) **D189**
serine endopeptidase [Crotalus mitchellii]

Sequence ID: AUS82518.1 204/233(88%) 213/233(91%) **N189**
serine endopeptidase [Crotalus lepidus]
Sequence ID: AUS82511.1 211/233(91%) 219/233(93%) **N189**
serine endopeptidase [Crotalus atrox]
Sequence ID: AUS82489.1 211/233(91%) 219/233(93%) **D189**

>Bnubes SVSP-4/Bnigro SVSP-4
IIGGDECNINEHRSLVLVNFDGFQCAGTLINQEWVLSAAHCDGKKMKLQFGLHSKNVPNKDKQT
RVAKEKFFCLSSKNYTKWDKDIMLIRLNRPVNNSTHIAPLSLPSNPPSVGSVCRIMGWGTISST
KVNLPDVPHCANINIIDYEVCRTAYPQYGLPATSRTLCAGILEGGKDTCVGDSGGPLICNGQFQ
GIVSWGSDVCGYIREPALYTKVLDYTDWIQSIIAGNTNATCPP

serine endopeptidase [Crotalus scutulatus] **D189**
Sequence ID: AUS82539.1 193/234(82%) 209/234(89%)
serine endopeptidase [Crotalus atrox] **D189**
Sequence ID: AUS82482.1 195/234(83%) 212/234(90%)
serine proteinase isoform 2 [Sistrurus catenatus edwardsi] **D189**
Sequence ID: ABG26968.1 195/235(83%) 210/235(89%)
serine endopeptidase [Crotalus scutulatus] **D189**
Sequence ID: AUS82539.1 193/234(82%) 209/234(89%)
serine endopeptidase [Crotalus lepidus] **D189**
Sequence ID: AUS82507.1 191/234(82%) 211/234(90%)

>Bnubes SVSP-5/Bnigro SVSP-5
VVGGDECNINEHRSLVLVYSDGIQCGGTLINQEWMLTAAHCDGKKMKLQFGLHSKNVPNKDKQT
RVPKEKFFCLSSKNYTKWDKDIMLIRLNHPVNNSTHIAPLSLPSNPPSQDTVCNIMGWGTISAS
KEIYPDVPHCANINILNNAVCRPIYSGLLEKSKTLCAGILEGGKDTCGGDSGGPLICNGQIQGI
LSVGGDPCALPHVPALYTKVFDYTEWIQSIIAGNTDAACLP

kallikrein-like serine protease [Trimeresurus gracilis] **D189**
Sequence ID: UVJ66759.1 213/233(91%) 219/233(93%)
serine proteinase isoform 11 [Sistrurus catenatus edwardsi] **D189**
Sequence ID: ABG26977.1 210/233(90%) 216/233(92%)
serine endopeptidase [Crotalus atrox] **D189**
Sequence ID: AUS82481.1 209/233(90%) 215/233(92%)
snake venom serine proteinase 11 iso X1 [Crotalus tigris] **D189**
Sequence ID: XP_039181657.1 207/233(89%) 216/233(92%)
snake venom serine proteinase 11 iso X2 [Crotalus tigris] **D189**
Sequence ID: XP_039181658.1 207/233(89%) 216/233(92%)
serine endopeptidase [Crotalus lepidus] **D189**
Sequence ID: AUS82510.1 206/233(88%) 214/233(91%)
serine endopeptidase [Crotalus mitchellii] **D189**
Sequence ID: AUS82517.1 207/233(89%) 213/233(91%)
serine endopeptidase [Crotalus scutulatus] **D189**
Sequence ID: AUS82557.1 206/233(88%) 214/233(91%)

>Bnubes SVSP-3/Bnigro SVSP-3
IIGGDECNINEHRFLVALYTFRSRRFHCSGTLINQEWVLTAAHCDRKNIRIKLGMHSTNVTNED
KQTRVPKEKFFCLSSKTYTKWDKDIMLIRLKRPVNKSIHIAPVSLPSNPPSLGSVCRIMGWGTI
SATKETYPEVPHCANINILDYEVCRGAFPWLPATSRTLCAGILEGGKDSCKGDSGGPLICNGQF
HGIVSWGGDPCALPRVPGLYTKVFDYTEWIQSIIAGNADATCPP

serine endopeptidase [Crotalus scutulatus] **D189**
Sequence ID: AUS82543.1 210/236(89%) 218/236(92%)

serine endopeptidase [Crotalus mitchellii] **D189**
Sequence ID: AUS82526.1 211/236(89%) 218/236(92%)
serine endopeptidase [Crotalus lepidus] **D189**
Sequence ID: AUS82501.1 211/236(89%) 218/236(92%)
serine endopeptidase [Crotalus atrox] **D189**
Sequence ID: AUS82487.1 210/236(89%) 217/236(91%)
serine endopeptidase [Crotalus cerastes] **D189**
Sequence ID: AUS82499.1 209/235(89%) 216/235(91%
